# MiR-27a-5p Increases Steer Fat Deposition Partly by Targeting Calcium-sensing Receptor (CASR)

**DOI:** 10.1038/s41598-018-20168-9

**Published:** 2018-02-14

**Authors:** Wucai Yang, Keqiong Tang, Yaning Wang, Linsen Zan

**Affiliations:** 1 College of Animal Science and Technology, Northwest A&F University, Yangling, Shaanxi 712100 China; 2 College of Veterinary Medicine, Northwest A&F University, Yangling, Shaanxi 712100 China

## Abstract

Castration increases fat deposition, improving beef quality in cattle. Here, the steer group exhibited a significantly higher intramuscular fat (IMF) content than the bull group. To determine the potential roles of microRNAs (miRNAs) in castration-induced fat deposition, differential expression patterns of miRNA in liver tissue were investigated in bulls and steers. A total of 7,827,294 clean reads were obtained from the bull liver library, and 8,312,483 were obtained from the steer liver library; 452 conserved bovine miRNAs and 20 novel miRNAs were identified. The results showed that the expression profiles of miRNA in liver tissue were changed by castration, and 12 miRNAs that were differentially expressed between bulls and steers were identified. Their target genes were majorly involved in the metabolic, PI3K-Akt, and MAPK signaling pathways. Furthermore, six differentially expressed miRNAs were validated by quantitative real-time PCR, and luciferase reporter assays verified that calcium-sensing receptor (CASR) was the direct target of miR-27a-5p. Meantime, we found that the expression level of CASR was significantly higher in steers than in bulls, and revealed that CASR gene silencing in bovine hepatocytes significantly inhibited triacylglycerol (TAG) accumulation and reduced secretion of very low density lipoprotein (VLDL). These results obtained in the liver indicate that miR-27a-5p may increase fat deposition partly by targeting CASR in steers.

## Introduction

When compared with intact males, castrated bulls exhibit lower growth rates and feed efficiencies, but increased carcass back fat^1,2^ and intramuscular fat (IMF) content^3–5^. Therefore, castration has been proposed as a means of enhancing meat quality, and the use of castrated males is now increasing because of their higher market value^6^. However, the mechanisms underlying differences in the fat deposition after castration are not well understood. The major sites where lipogenesis generally takes place are the liver and adipose tissue^7^. The liver is the central organ for uptake, oxidation, and metabolic conversion of non-esterified fatty acids^7–9^. Moreover, it has enzymatic capacity for de novo lipogenesis, cytoplasmic triacylglycerol stores, and the synthesis of fatty acids from glucose and other non-lipid precursors^7,9^. The relative importance of these functions depends on species differences^10^. A well recognized and major metabolic difference between ruminants and non-ruminants is the failure of carbon from glucose to contribute to fatty acid synthesis within the tissues of ruminants^10,11^. As a result, only modest amounts of de novo lipogenesis occurs in the liver of ruminants compared to that of non-ruminants^7^, but it still plays an important role in ruminant lipid metabolism^12^.

With the development of high-throughput sequencing technologies, comparative analysis of liver transcriptome has been performed to explore potential candidate genes which affect fat deposition in bovines^13^, and many genes characterized by an extreme capacity for IMF deposition have been identified^13^. MicroRNAs (miRNAs) are small regulatory molecules involved in the posttranscriptional regulation of gene expression, and previous studies have clearly demonstrated that miRNAs play an important role in various biological processes, including development, differentiation, and metabolism^13^. Since the first fat metabolism-related miRNA, miR-14, was discovered in *Drosophila*^14^, increasing evidence has indicated that miRNAs are involved in regulating adipogenesis and lipid metabolism^15–18^. For example, miR-130a has been reported as an important regulator of adipogenesis in human primary preadipocytes and in the 3T3-L1 cell line, targeting specific adipogenic factors^19^; miR-146b accelerates adipocyte differentiation by negatively regulating sirtuin 1 (SIRT1) in 3T3L1 cells^20^. Moreover, miRNAs have been demonstrated to play vital roles in modulating lipid metabolism in the liver^21,22^. MiRNA-21 regulates hepatic glucose metabolism by targeting forkhead box O1 (FoxO1)^23^; miRNA-122 is expressed only in the liver, which inhibits the triglyceride synthesis by targeting 1-acylglycerol-3-phosphate O-acyltransferase 1 and diacylglycerol acyltransferase 1^24^; overexpression of miR-212-5p significantly decreases triglyceride synthesis in primary hepatocytes^25^. Previous studies have suggested that miRNA expression profiles in skeletal and adipose tissue were significantly altered by castration^22,26^. However, there are no reports of lipid metabolism-related miRNA expression profiles in liver. Specifically, no studies have addressed whether the miRNA expression patterns in bovine liver could be changed by testosterone loss due to castration.

Calcium-sensing receptor (CASR) gene is a G-protein-coupled receptor that plays a critical role in the development of obesity^27^. Using antagonists or siRNA of CASR demonstrated that CASR can elevate the preadipocyte proliferation and increase fat accumulation by mediating antilipolytic pathways concomitantly with other relevant changes in lipolysis-regulating enzymes^27,28^. CASR not only functions in adipose tissue^29,30^, but also directly and indirectly affects peripheral organs such as the liver^31^. Recent studies indicated that CASR plays a critical role in the regulation of triglyceride production by affecting the activity of the microsomal triglyceride transfer protein^32,33^.

Here, we first compared the miRNA expression in liver tissue between bulls and steers to identify novel lipid metabolism-related and differentially expressed miRNAs. We then used bioinformatics methods to predict the target genes of these miRNAs, which may be involved in lipid metabolism. Quantitative real-time PCR and luciferase reporter assays were used to validate and identify lipid metabolism-related miRNAs and their predicted target genes. The aim of this research was to gain new insight into lipid metabolism-related miRNAs in cattle, which will improve understanding of the mechanism of fat deposition after castration.

## Results

### IMF content and deep sequencing of bovine short RNAs

**Figure 1:**
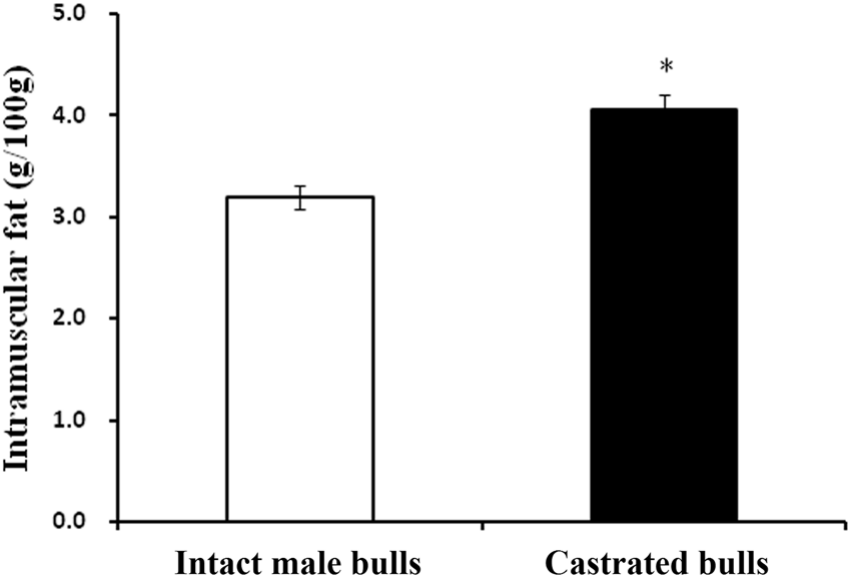
Effects of castration on bovine intramuscular fat content. Values are presented as the means ± S.E.M. “*”Represents significant differences *P* < 0.05.

**Figure 2:**
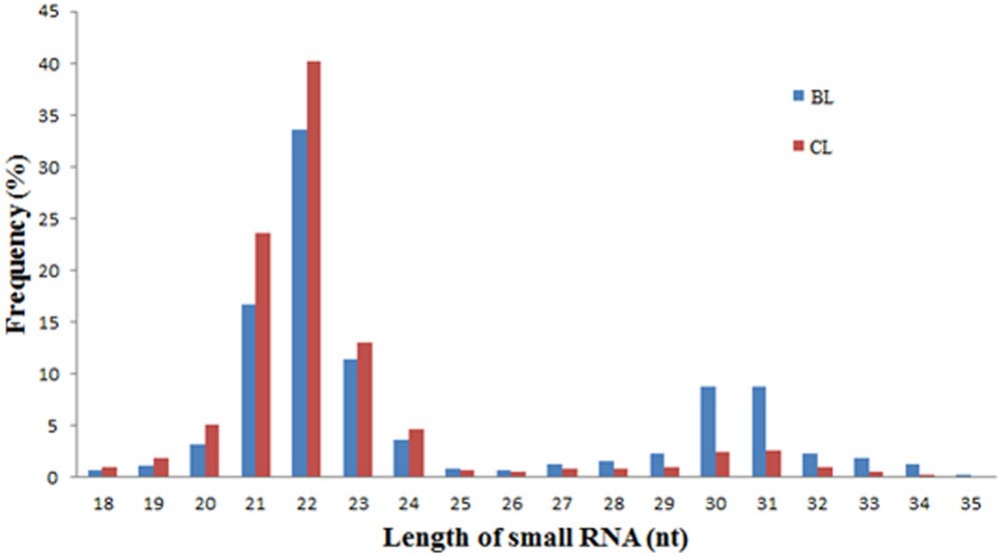
Length distribution of small RNAs in BL and SL libraries.

The SL group exhibited a significantly higher IMF content than the BL group 24 months post-castration (P < 0.05; Fig. 1). Solexa sequencing provided 8,413,121 and 7,924,174 reads of 3 nt-35 nt from the SL and BL libraries, respectively. A total of 8,312,483 and 7,827,294 clean reads of 18–30 nt were obtained by removing low-quality reads, adaptors, and insufficient tags and sequences (Table 1). The trend of the small RNA size distribution (18 nt-29 nt) between bulls and steers was similar, and most reads ranged from 21 to 24 nt **(**Fig. 2). In total, 5,245,556 reads were matched to the bovine genome in the SL library, and 4,695,270 reads were from the BL library. Furthermore, 67.97% of the sequencing reads were known miRNAs in the SL library, and 85.36% in the BL library (Figure S1).

**Table 1:** Summary of small RNA sequencing date.

Type	BL		SL	
	Count	%	count	%
Total-read	8413121		7924174	
N% > 10%	24	0.00%	22	0.00%
Low quality	784	0.01%	581	0.01%
Adaptor 3-null or Insert-null	96301	1.14%	92816	1.17%
Adaptor 5-contaminants	411	0.00%	273	0.00%
PolyA/T/G/C	3118	0.04%	3188	0.04%
Clean-reads	8312483	98.80%	7827294	98.78%

### Identification of known and novel bovine miRNAs

In total, 472 unique miRNA genes were identified, including 20 novel and 452 known miRNAs (Table 2). Furthermore, 384 miRNAs overlapped between these two libraries, and 416 and 440 miRNAs, including 20 and 18 novel miRNAs, were identified in the SL and BL libraries, respectively (Table 2).

**Table 2:** Numbers of known and novel miRNAs in castrated and intact bulls.

Group	Novel miRNAs	Known miRNAs	Total
BL	18	398	416
SL	20	420	440
Total	20	452	472

### MiRNAs differentially expressed between intact and castrated bull liver

We compared the bull and steer liver libraries and found that these two tissues had different miRNA expression profiles. In these two libraries, bta-let-7f, bta-miR-122, bta-miR-148a, bta-miR-192, bta-miR-21-5p, bta-miR-26a/c, and bta-miR-30a-5p were the dominantly expressed miRNAs (Table S2). All the novel miRNAs (including 14 co-expressed, 2 steer-specific and 4 bull-specific) had lower expression levels than those of the conserved miRNAs (Table S2). Furthermore, one significantly up-regulated miRNA and 11 significantly down-regulated miRNAs were identified by comparing the expression levels of miRNAs between steers and bulls (Table S3 and Figure S2).

### Target gene prediction, GO enrichment, and KEGG pathway analysis

In total, 3235 target genes were predicted by TargetScan for those 12 differentially expressed miRNAs. The GO assignments showed that these target genes were involved in the molecular function, cellular component, and biological process categories (Table S4). Moreover, the KEGG pathway annotation found 6177 target genes annotated for 276 biological processes with P ≤ 1 (Table S5). The metabolic, PI3K-Akt signaling and MAPK signaling pathways enriched the most genes (Table S5).

### Validation of predicted miRNA targets

**Figure 3:**
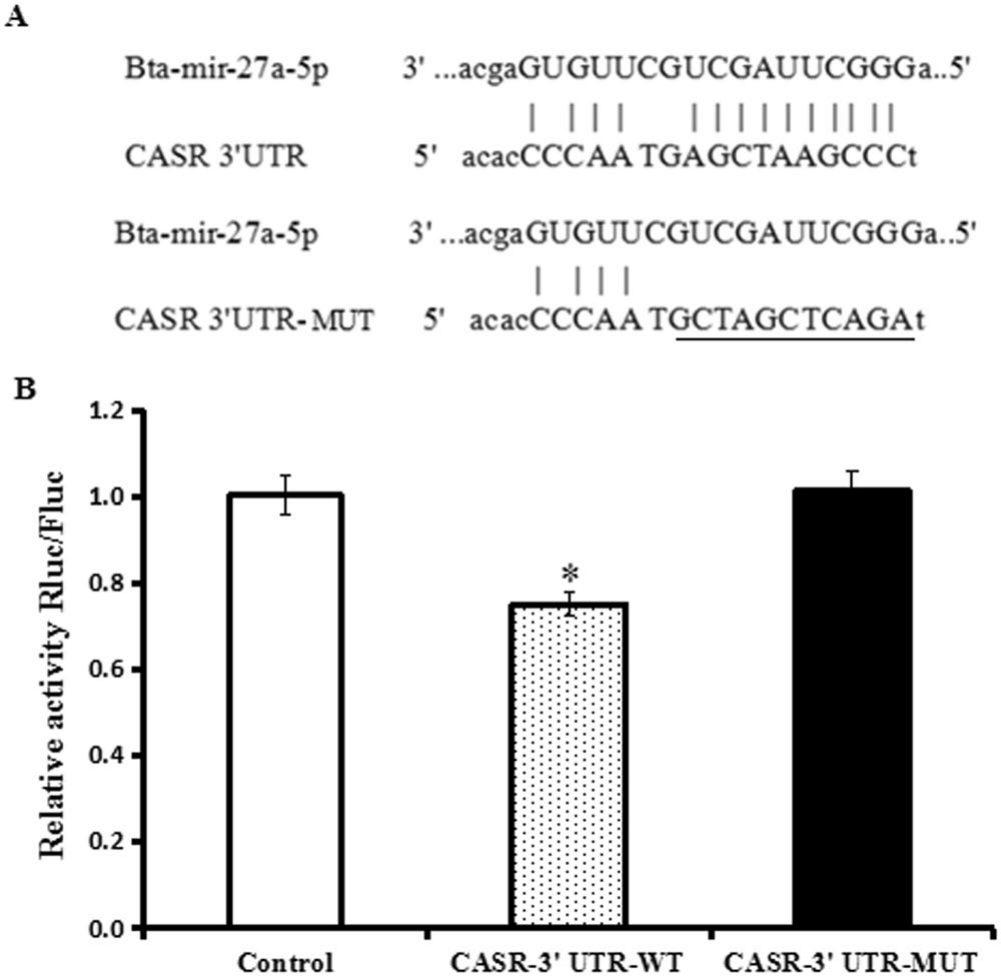
(**A**) Sequence alignment of miR-27a-5p and 3′-UTR of CASR using the TargetScan and miRDB algorithms. (**B**) pmirGLO-CASR -3′UTR-WT or a mutant CASR-3′UTR-MUT was co-transfected into 293 T cells with miR-27a-5p mimics. The assay showed that the luciferase activity in the CASR -3′ UTR-WT group was significantly decreased but was increased when compared with the mutant groups. Values are presented as the means ± S.E.M. “*”Represents significant differences *P* < 0.05.

Whether the CASR gene is a direct target of miR-27a-5p was verified by a dual-luciferase reporter system. The miR-27a-5p mimics significantly decreased the normalized luciferase activity by 23.0% compared to the negative control (P < 0.05, Fig. 3), and the luciferase activity in the CASR -3′ UTR- MUT group rose to normal levels again (Fig. 3).

### Validation of miRNA and CASR gene expression

**Figure 4:**
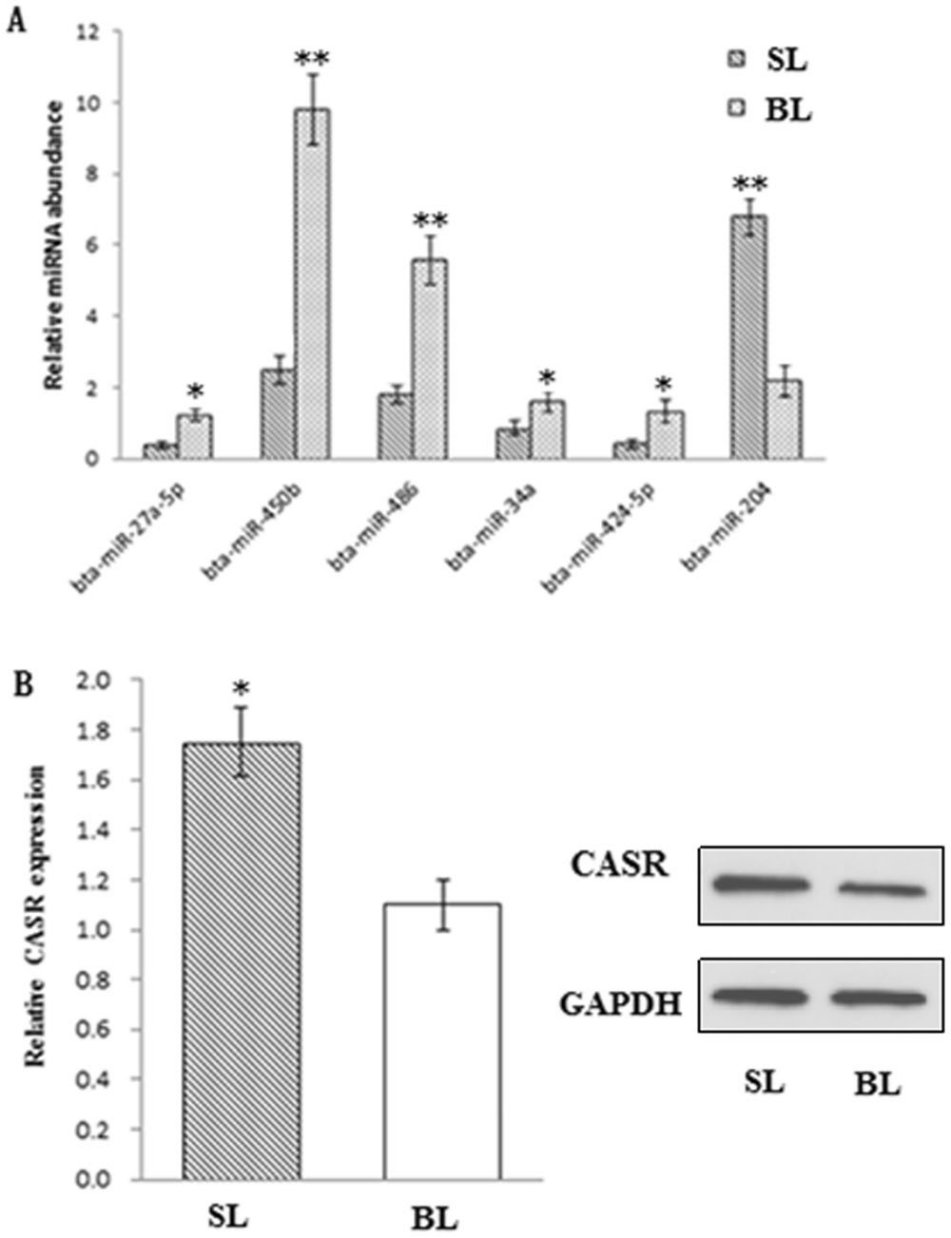
(**A**) The expression of miRNAs in BL and SL. (**B**) The mRNA and protein expression of CASR gene in BL and SL. Values are presented as the means ± S.E.M. “*”Represents significant differences *P* < 0.05; “**”represents significant differences *P* < 0.01.

Six miRNAs were validated in bull and steer liver. The expression levels of bta-miR-27a-5p, bta-miR-486, bta-miR-450b, bta-miR-424-5p and bta-miR-34a were significantly higher in bulls than in steers, and the bta-miR-204 expression level was lower in bulls (Fig. 4A), which is consistent with the results of RT-qPCR and Solexa sequencing. Moreover, the mRNA and protein expression levels of the miR-27a-5p-targeted gene CASR were significantly higher in steers than in bulls (Fig. 4B).

### CASR gene silencing inhibits TAG accumulation and reduces secretion of VLDL

**Figure 5:**
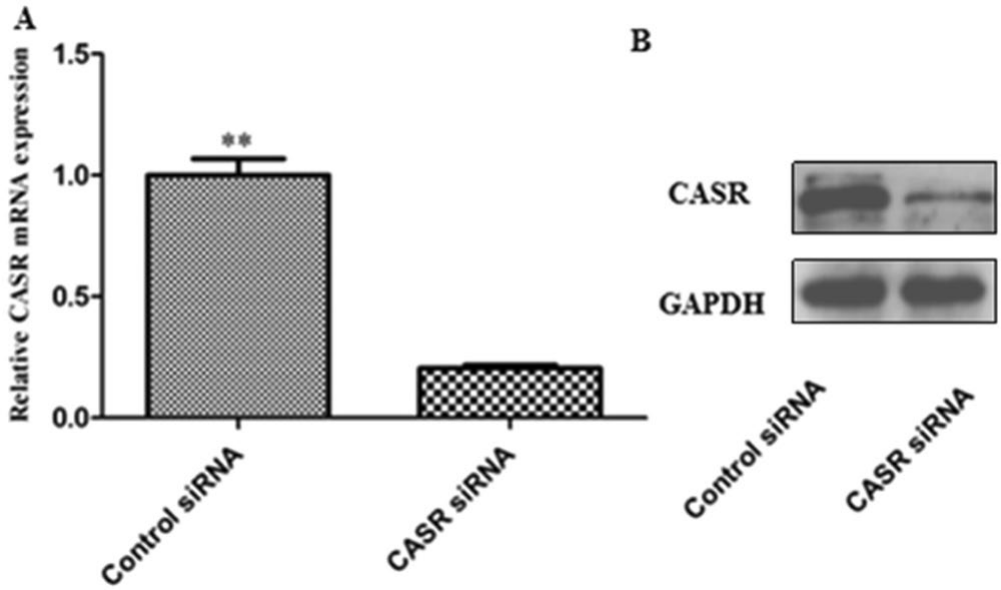
Validation of CASR knockdown. (**A**) CASR mRNA levels in bovine hepatocytes transfected with control or CASR siRNA. (**B**) A representative western blot of CASR protein in bovine hepatocytes transfected with control or CASR siRNA. Values are presented as the means ± S.E.M. “**”Represents significant differences *P* < 0.01.

**Figure 6:**
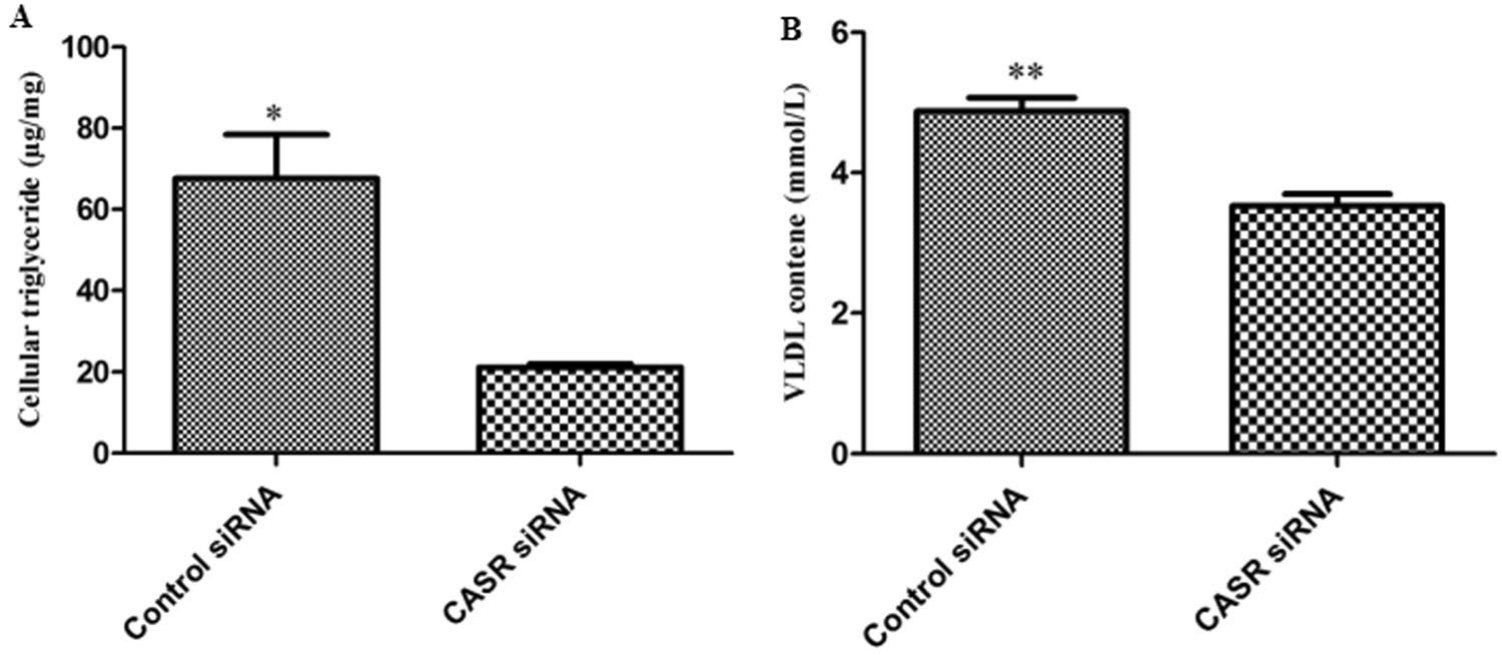
Changes in cellular TAG and extracellular VLDL after transfected with control or CASR siRNA in bovine hepatocytes. (**A**) TAG content. (**B**) Extracellular VLDL content. Values are presented as the means ± S.E.M. “*”Represents significant differences *P* < 0.05; “**”represents significant differences *P* < 0.01.

To investigate the roles of CASR in lipid metabolism in hepatocytes, CASR silencing assay were performed by using specific siRNA. The most appropriate transfection concentration of siRNA in bovine hepatocytes was determined to be 50 nM and high knock down efficiency (Fig. 5A and B). CASR gene silencing significantly inhibited cellular TAG accumulation (Fig. 6A) and reduced secretion of VLDL in culture medium compared with the control cells (Fig. 6B).

## Discussion

MicroRNAs are small RNA molecules of approximately 18–23 nucleotides in length that play a vital regulatory role via a post-transcriptional mechanism. An association of miRNAs with fat synthesis induced by castration has been reported in skeletal and adipose tissue in ruminants^22,26^. However, the miRNA expression profile induced by castration in liver tissue and its effects on fat deposition are still unrevealed in bovine. We therefore compared the expression of miRNAs in the liver tissue between bulls and steers. In this study, the trend of small RNA size distribution (18 nt-29 nt) between bull and steer was similar, and most reads ranged from 21 to 24 nt. This is consistent with a previous study in which 22 nt sequences were significantly more common than other lengths in cattle adipose and muscular tissues, and in pig back fat^17,34,35^, but not consistent with previous studies on miRNA in testes and ovaries in bovine and maize^36,37^. This result may be due to the vast differences in experimental approaches, species, and tissues.

In our datasets, bta-miR-204 was the only miRNA with decreased expression in the bull liver (BL) library compared to the steer liver (SL) library. Gao *et al*.^25^ found that SIRT1 is the direct target gene of miR-204-5p^38^, and SIRT1 has been demonstrated to promote the expression of adipose triglyceride lipase gene and glycerol release^39^. Moreover, bta-miR-204 was determined to be present in the reproductive organs and was found to be significantly associated with semen quality traits in bovines^40^. These results indicated that bta-miR-204 may be involved in lipid metabolism in cattle and may be regulated by testosterone. In this study, bta-miR-27a-5p was expressed in high abundance, followed by bta-miR-486, bta-miR-450b, bta-miR-424-5p and bta-miR-34a in the BL library compared to the SL library. Previous studies reported that miR-486 regulates lipid metabolism through the PI3K-Akt-signaling pathway by targeting the phosphorylation of Akt and tensin homolog and FoxO1^38,41,42^. MiR-34a regulates the activity of AMP kinase, a known regulator of energy metabolism, by suppressing SIRT1^43^, and has been reported to emerge as a specific miRNA modulated lipid metabolism in the liver^44^. MiR-450b-5p significantly arrested the growth of rhabdomyosarcoma and promoted its myogenic differentiation^45^, and miR-424-5p has been widely studied due to its possible involvement and important role in cancer metastasis or tumor suppression^46,47^, but the function of bta-miR-424-5p and bta-miR-450b-5p in lipid metabolism has not been determined. Furthermore, our results showed that the metabolic pathway, the PI3K-Akt signaling pathway and the MAPK signaling pathway enriched the most genes. Previous studies showed that the MAPK and PI3K/Akt signaling pathways are closely related to lipid metabolism and the secretory activity of liver cells^41,48,49^.

Bta-miR-27a-5p was expressed at low abundance in the SL library compared to expression in the BL library. A previous study found that miR-27a regulated many lipid metabolism-related transcription factors, including RXRα, PPARγ, FASN, SREBP1, and SREBP2 in human hepatoma cells^50^. Over-expressed microRNA-27a suppressed fat synthesis during rat hepatic stellate cell activation^51^, and down-regulated the key enzyme genes expression involved in de novo lipid synthesis^52^. Recently, Shirasaki *et al*. demonstrated that the lipid transporter ABCA1 is a target of miR-27a^50^, and that the knockdown of ABCA1 in rat hepatoma cells increased triglyceride secretion to the culture medium and decreased the cellular levels of FFA^53,54^. Here, our results confirmed that the calcium-sensing receptor (CASR) gene was verified to be a direct target gene of miR-27a-5p. Meantime, we found that the expression of the CASR gene was found to be significantly higher in steers than in bulls, and CASR gene silencing in bovine hepatocytes significantly inhibited TAG accumulation and reduced secretion of VLDL. Researches in ruminants, especially in cattle, reported that hepatic TAG synthesis is the result of various pathways of lipid metabolism, including de novo synthesis of fatty acids and secretion of triacylglycerols via VLDL^55–57^. CASR gene is a G-protein-coupled receptor that plays a critical role in lipid metabolism^23^. Recent studies found that CASR has higher expression in obese patients^33^, and reported that CASR promotes TAG production via affecting the activity of the microsomal triglyceride transfer protein for VLDL secretion in liver^27–29,31,32^. Furthermore, recent studies indicated that CASR stimulation significantly elevated TAG synthesis in hepatocytes by facilitating the uptake of long-chain fatty acids^33,58,59^. These results indicate that miR-27a-5p may increase fat deposition partly by targeting CASR in steers via facilitating the production of TAG and the secretion of TAG as VLDL in the liver^31–33,55–59^.

## Conclusions

In this study, we firstly found that lipid metabolism-related miRNAs differentially expressed in liver tissue between bull and steer. Furthermore, six differentially expressed miRNAs were validated by qRT-PCR, and the results supported CASR as a miR-27a-5p direct target gene. Meantime, we found that the expression of the CASR gene was found to be significantly higher in steers than in bulls, and CASR gene silencing in bovine hepatocytes significantly inhibited TAG accumulation and reduced secretion of VLDL. These results obtained in the liver indicate that miR-27a-5p may increase fat deposition by partly targeting CASR in steers. Our study provides a new clue for understanding the changes in the carcass backfat and intramuscular fat deposition caused by castration-induced testosterone deficiency.

## Materials and Methods

### Ethics statement

This study was conducted in strict accordance with the Regulations for the Administration of Affairs Concerning Experimental Animals (Ministry of Science and Technology, China, revised 2004). The protocol was approved by the Committee on the Ethics of Animal Experiments of the Laboratory Animals of Northwest A&F University (Permit Number: AE189056). All surgery was performed under sodium pentobarbital anesthesia, and all efforts were made to minimize suffering.

### Sample collection and RNA extraction

Six Qinchuan bull calves from one experimental farm were randomly selected to be unrelated for at least three generations, and three of these six bull calves were castrated at 6 months old. The sternomandibularis muscle tissue of each animal was sampled, and the intramuscular fat (IMF) tissue was quickly dissected. IMF content was analyzed as previously described^60^. Liver tissues were collected immediately from the bulls and steers, which were slaughtered at 24 months old. All tissue samples were promptly frozen in liquid nitrogen and stored at -80 for further analysis. The total RNA was extracted from the collected tissues using Trizol reagent (TaKaRa, Dalian, China). The extracted RNAs from the same group were pooled prior to constructing the indexed libraries for Illumina sequencing.

### Sequencing data analysis

Raw reads obtained from Illumina sequencing were cleaned of poor quality reads. The clean reads were screened against and mapped to the latest bovine genome assembly using the program SOAP^61^, and screened against the GenBank non-coding RNA database (http://blast.ncbi.nlm.nih.gov/) and the Rfam database (http://www.sanger.ac.uk/resources/databases/rfam.html) to remove non-coding RNA. Subsequently, the small RNA reads were searched against mRNA introns and exons to identify degraded mRNA fragments in the small RNA tags. The remaining reads were aligned against the known bovine miRNA precursors and mature miRNAs deposited in miRBase 18.0^62^ to identify the bovine conserved miRNAs. To discover potential novel miRNA precursor, unique sequences which had more than 10 hits to the bovine genome or matched other non-coding RNAs were removed. The flanking sequences (150 nt upstream and downstream) of each unique sequence were then extracted for secondary structure analysis with Mfold (http://www.bioinfo.rpi.edu/applications/mfold) and then evaluated by Mireap (http://sourceforge.net/projects/mireap/). Those sequences residing in the stem region of the stem-loop structure and ranging between 20–22 nt with free energy hybridization lower than 220 kcal/mol were considered novel miRNAs^63^.

### MicroRNA expression analysis

A comparison of the expression of known miRNAs between steer liver (SL) and bull liver (BL) was conducted to find tissue-enriched miRNAs. The procedures were as follows: (1) Normalize the expression of miRNAs in SL and BL. Normalized expression (NE) = actual miRNA count/total count of clean reads. (2) Calculate fold change and P-value from the normalized expression.

Stem-loop real-time reverse transcription polymerase chain reaction (RT-PCR) with SYBR Green was used for the validation of miRNA expression^64^, and 5S gene was used as an endogenous control. For evaluation of relative CASR expression, β-actin was used as the internal control. The primers of tested miRNAs, CASR, and the control gene are listed in Table S1. All reactions were carried out in triplicate. The analysis of relative miRNA expression used the 2^−ΔΔCT^ method.

### Target gene prediction, gene ontology (GO), and pathway analysis

The target sites in the 3′ untranslated regions of the gene transcripts for differentially expressed miRNAs were predicted using miRanda^65^ and RNAhybrid^66^ software. Gene ontology (GO) is an international standardized classification system for gene function. It uses a controlled vocabulary set to comprehensively describe the properties of genes and gene products. The basic unit of GO is the GO term, each of which belongs to one type of ontology. The following GOs were detected: molecular function, cellular component, and biological process. Moreover, GO enrichment analysis was used on the common target genes of differentially expressed miRNAs predicted by both miRanda and RNAhybrid. This analysis provides all GO terms that are significantly enriched in the predicted target gene compared with the reference gene background, as well as the genes corresponding to certain biological functions.

This method first maps all the target genes of differentially expressed miRNAs to the GO terms in the database (http://www.geneontology.org/), calculates the gene numbers for each term, and then uses a hypergeometric test to find significantly enriched GO terms in target genes compared with the reference gene background.

KEGG pathway analysis identifies significantly enriched metabolic pathways or signal transduction pathways in target genes by comparing them with the whole reference gene background. The formula is the same as that used in GO analysis. Target genes with P < 0.05 are considered significantly enriched. The KEGG analysis identifies the main pathways in which the target gene candidates are involved in.

### miRNA target validation using dual luciferase reporter assay

The CASR -3′UTR-WT or MUT were cloned into the pmirGLO Dual-Luciferase miRNA Target Expression Vector (Promega) using the Xho1 and Not1 restriction sites. The 293 T cells were cultured in 24-well plates 1 day before transfection. Approximately 0.16 ug of CASR -3′ UTR-WT or -MUT vectors were cotransfected with 5 pmol miR-27a-5p mimics or negative control (NC) into 293 T cells using Lipofectamine 2000 reagent (Invitrogen). After 48 hours of transfection, luciferase activity was measured using the Dual-Luciferase Reporter Assay System (Promega Corporation) according to the manufacturer’s instructions. Firefly luciferase activity was normalized to the activity of Renilla luciferase.

### Cell culture

Liver tissues were obtained under sterile conditions from neonatal calf. The hepatocytes were isolated by collagenase IV perfusion method as described by Li *et al*.^67^ and Shi *et al*.^68^. The hepatocyte was seeded into a six-well tissue culture plate (2 mL per well) at 1 × 10^6^ cells/mL and incubated at 37 °C in 5% CO2.

### siRNA transfection

Cells were seeded in 6-well plates and when grown to 70% confluence, siRNAs were transfected according to the standard protocol at a final concentration of 50 nM. Briefly, 1.32 μl X-tremeGENE HP DNA transfection reagent (Roche) and 0.66 μg siRNA were diluted in Opti-MEM (Gibco) respectively for 10 minutes. The two mixtures were then mixed for another 15 minutes at room temperature to allow to form transfection reagent-siRNA complexes. The complex mixture was then added to the cell culture medium. Cells were replaced with fresh complete growth medium 8 h later. The siRNA transfection was performed in triplicate wells and the experiment was performed with 3 repeats.

### Western blot

Cells were collected and lysed in radioimmunoprecipitation assay buffer (Solarbio, Beijing, China) supplemented with phenylmethanesulfonyl fluoride (Pierce, Rockford, IL) after treatment for 48 h. Total protein concentrations were determined by bicinchoninic acid assays (Sangon Biotech). Proteins were separated on 10% polyacrylamide gels and transferred onto polyvinylidene fluoride membranes (Millipore, Bedford, MA). After overnight blocking with 5% dried nonfat milk in PBS containing 0.1% Tween 20, membranes were incubated with mouse anti-CASR polyclonal antibody (1:500; Abcam, Cambridge, MA, US) and mouse anti-GAPDH monoclonal antibody (1:1000; Santa Cruz Biotechnology). Membranes were then washed 3 times with PBS-Tween and incubated for 1 h at room temperature with 3,500-fold diluted horseradish peroxide-labeled mouse anti-rabbit IgG (Santa Cruz Biotechnology). Immunoreactive bands were detected using the enhanced chemiluminescence detection kit (Amersham Biosciences, Little Chalfont, UK) and scanned on a chemiluminescent imaging system (MFChemiBIS3.2, DNR Bio-Imaging Systems Ltd., Jerusalem, Israel).

### TAG assay

The levels of cellular TAG in bovine hepatocytes were assayed using the tissue triglyceride assay kit (Applygen Technologies, Beijing, China). Total protein concentrations were determined by the BCA assay (Sangon Biotech). All of the experiments were performed according to the manufacturer’s recommended protocol. The values obtained were normalized to the total cellular protein content and were expressed as micrograms per milligram of protein.

### VLDL assay

Culture medium was collected and frozen at −20 °C after treatment for 48 h. The levels of VLDL in culture medium were assayed using the VLDL assay kit (Shanghai Enzyme-linked Biotech, Shanghai, China) according to manufacturer’s instructions.

## Electronic supplementary material

Supplementary information
