# Supplementary material for: MiR-27a-5p Increases Steer Fat Deposition Partly by Targeting Calcium-sensing Receptor (CASR)
Source: Sci Rep. 2018 Feb 14;8:3012. doi: 10.1038/s41598-018-20168-9 (PMC5813002; doi:10.1038/s41598-018-20168-9)
Supplement: Supplementary file 1 — Supplementary information [file 41598_2018_20168_MOESM1_ESM.pdf]

# Title: MiR-27a-5p Increases Steer Fat Deposition Partly by Targeting Calcium-sensing Receptor (CASR)

Wucai Yang, Keqiong Tang, Yaning Wang, Linsen Zan

**Supplementary figures:**

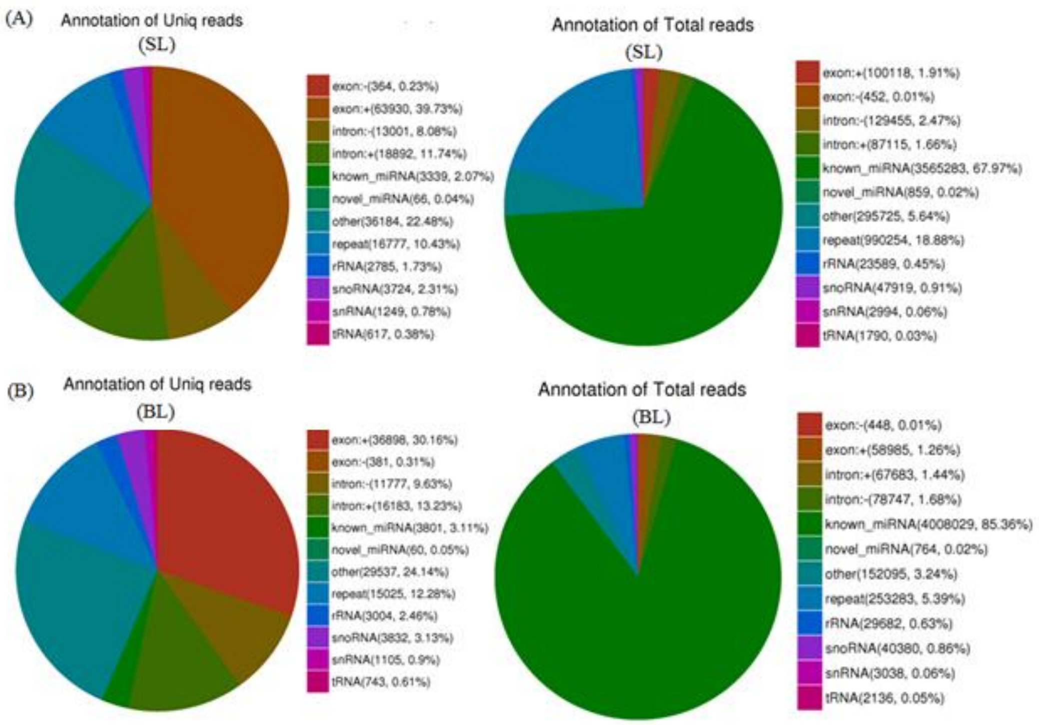


**Figure S1.** Distribution of the genome-mapped sequence reads in BL (A) and SL (B) small RNA libraries.

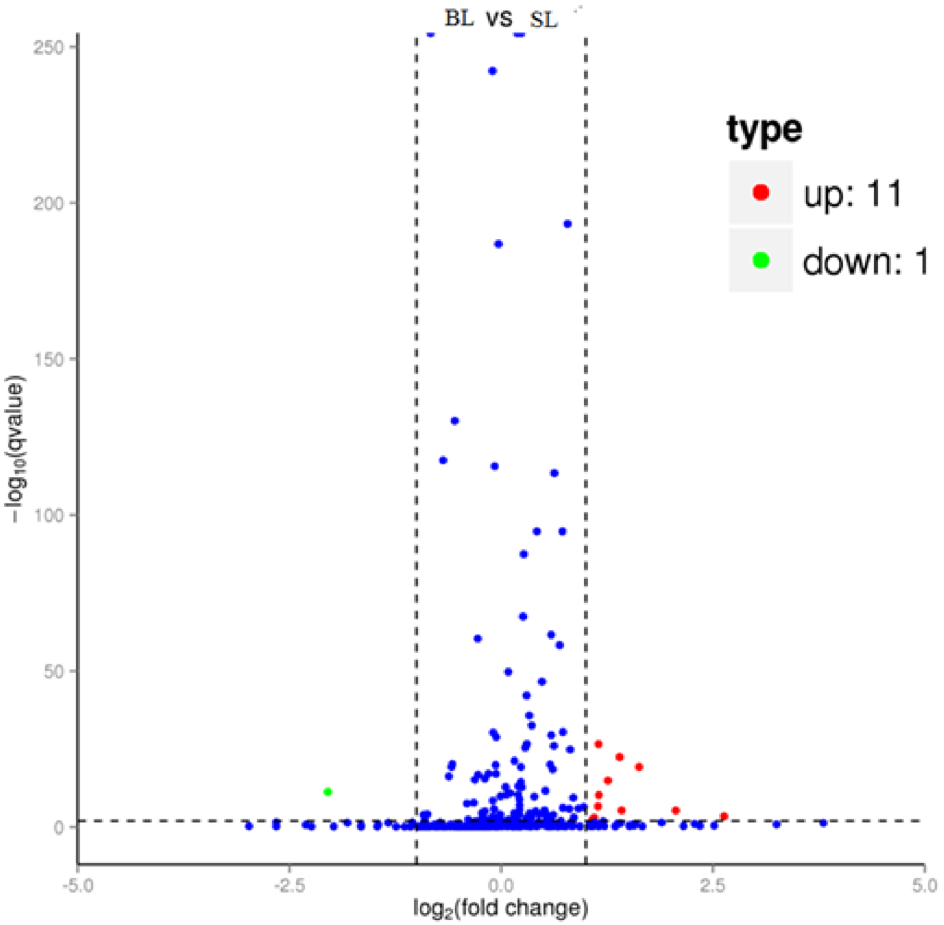


**Figure S2.** The differential expression of bovine miRNAs between BL and SL tissue were shown. Note: Each point in the figure represents a miRNA. Red points represent up-expressed miRNAs; blue points represent equally-expressed miRNAs; green points represent down-expressed miRNA.

## Supplementary tables:

**Table S1** Primers for quantitative real-time PCR

| Genes | Primer sequence | $T_{ann}$ (℃) |
|---|---|---|
| bta-miR-27a-5p | F:AGGGCTTAGCTGCTTGTGAGCA | 65 |
| bta-miR-450b | F:GGCTTTTGCAATATGTTCCTGAATA | 65 |
| bta-miR-486 | F:CGTCCTGTACTGAGCTGCCCCGAG | 65 |
| bta-miR-34a | F:TGGCAGTGTCTTAGCTGGTTGT | 65 |
| bta-miR-424-5p | F:CAGCAGCAATTCATGTTTTGA | 65 |

| | | |
|---|---|---|
| bta-miR-204 | F:TTCCCTTTGTCATCCTATGCCT | 65 |
| 5S | F:ACTCTTAGCGGTGGATCACTC<br>R:AGTGACGCTCAGAC AGGCATA | 65 |
| β-actin | F:CACCAACTGGGACGACAT<br>R:ATACAGGGACAGCACAGC | 65 |
| CASR | F:AGAGGACGGCTCCATAGTGTT<br>R:CCATCAGGACATTCCACACAC | 65 |

**Table S2 Expression levels of all miRNAs in BL and SL small RNA libraries**

| sRNA.readcount | SL.readcount | BL.readcount | SL.tpm |
|---|---|---|---|
| bta-let-7a-3p | 81 | 114 | 21.90587021 |
| bta-let-7a-5p | 39463 | 50105 | 10672.48588 |
| bta-let-7b | 13275 | 19575 | 3590.128728 |
| bta-let-7c | 4123 | 5561 | 1115.035838 |
| bta-let-7d | 1868 | 2511 | 505.1872289 |
| bta-let-7e | 1078 | 1684 | 291.5373837 |
| bta-let-7f | 117147 | 123596 | 31681.56762 |
| bta-let-7g | 94599 | 99118 | 25583.62241 |
| bta-let-7i | 41966 | 46970 | 11349.40431 |
| bta-miR-1 | 1629 | 2159 | 440.5513897 |
| bta-miR-100 | 27729 | 36175 | 7499.109567 |
| bta-miR-101 | 39069 | 70173 | 10565.9314 |
| bta-miR-103 | 9409 | 15696 | 2544.596701 |
| bta-miR-105a | 0 | 1 | 0 |
| bta-miR-106a | 3 | 23 | 0.811328526 |
| bta-miR-106b | 234 | 664 | 63.28362504 |
| bta-miR-107 | 887 | 2082 | 239.8828009 |
| bta-miR-10a | 2375 | 4617 | 642.3017498 |
| bta-miR-10b | 645 | 775 | 174.4356331 |
| bta-miR-1185 | 0 | 1 | 0 |
| bta-miR-1197 | 2 | 5 | 0.540885684 |
| bta-miR-122 | 1079632 | 1163763 | 291978.7464 |
| bta-miR-1224 | 0 | 2 | 0 |
| bta-miR-1246 | 10 | 18 | 2.70442842 |
| bta-miR-1248 | 44 | 11 | 11.89948505 |
| bta-miR-125a | 2737 | 4280 | 740.2020587 |
| bta-miR-125b | 7377 | 11100 | 1995.056846 |
| bta-miR-1260b | 4 | 10 | 1.081771368 |
| bta-miR-126-3p | 52534 | 53952 | 14207.44426 |
| bta-miR-126-5p | 1252 | 2266 | 338.5944382 |
| bta-miR-127 | 1267 | 3004 | 342.6510809 |
| bta-miR-1271 | 151 | 364 | 40.83686915 |
| bta-miR-1277 | 0 | 2 | 0 |
| bta-miR-128 | 532 | 839 | 143.875592 |

| | | | |
|---|---|---|---|
| bta-miR-129 | 10 | 10 | 2.70442842 |
| bta-miR-1291 | 0 | 1 | 0 |
| bta-miR-129-3p | 3 | 3 | 0.811328526 |
| bta-miR-1296 | 5 | 14 | 1.35221421 |
| bta-miR-1298 | 0 | 1 | 0 |
| bta-miR-1301 | 0 | 2 | 0 |
| bta-miR-1306 | 13 | 33 | 3.515756947 |
| bta-miR-1307 | 225 | 453 | 60.84963946 |
| bta-miR-130a | 51 | 76 | 13.79258494 |
| bta-miR-130b | 1 | 3 | 0.270442842 |
| bta-miR-132 | 7 | 14 | 1.893099894 |
| bta-miR-133a | 81 | 136 | 21.90587021 |
| bta-miR-133b | 4 | 4 | 1.081771368 |
| bta-miR-133c | 5 | 1 | 1.35221421 |
| bta-miR-134 | 8 | 21 | 2.163542736 |
| bta-miR-1343-3p | 12 | 41 | 3.245314104 |
| bta-miR-135a | 3 | 2 | 0.811328526 |
| bta-miR-136 | 58 | 82 | 15.68568484 |
| bta-miR-137 | 1 | 4 | 0.270442842 |
| bta-miR-138 | 1 | 7 | 0.270442842 |
| bta-miR-1388-3p | 14 | 18 | 3.786199789 |
| bta-miR-1388-5p | 91 | 170 | 24.61029863 |
| bta-miR-139 | 2728 | 4451 | 737.7680731 |
| bta-miR-140 | 6169 | 9311 | 1668.361893 |
| bta-miR-141 | 48 | 40 | 12.98125642 |
| bta-miR-142-3p | 168 | 162 | 45.43439746 |
| bta-miR-142-5p | 2101 | 3451 | 568.2004111 |
| bta-miR-143 | 80820 | 149955 | 21857.19049 |
| bta-miR-1434-3p | 0 | 1 | 0 |
| bta-miR-1434-5p | 1 | 2 | 0.270442842 |
| bta-miR-144 | 11 | 45 | 2.974871262 |
| bta-miR-145 | 4519 | 10989 | 1222.131203 |
| bta-miR-1468 | 526 | 482 | 142.2529349 |
| bta-miR-146a | 1576 | 2918 | 426.2179191 |
| bta-miR-146b | 727 | 1121 | 196.6119462 |
| bta-miR-147 | 19 | 24 | 5.138413999 |
| bta-miR-148a | 1006677 | 890539 | 272248.5889 |
| bta-miR-148b | 3689 | 5427 | 997.6636443 |
| bta-miR-149-5p | 7 | 16 | 1.893099894 |
| bta-miR-150 | 327 | 683 | 88.43480935 |
| bta-miR-151-3p | 1906 | 1639 | 515.4640569 |
| bta-miR-151-5p | 359 | 621 | 97.08898029 |
| bta-miR-152 | 6151 | 12964 | 1663.493921 |
| bta-miR-153 | 1 | 0 | 0.270442842 |

| | | | |
|---|---|---|---|
| bta-miR-154a | 0 | 1 | 0 |
| bta-miR-154b | 8 | 11 | 2.163542736 |
| bta-miR-154c | 131 | 388 | 35.42801231 |
| bta-miR-155 | 268 | 336 | 72.47868167 |
| bta-miR-15a | 131 | 299 | 35.42801231 |
| bta-miR-15b | 73 | 208 | 19.74232747 |
| bta-miR-16a | 2484 | 5020 | 671.7800196 |
| bta-miR-16b | 757 | 1818 | 204.7252314 |
| bta-miR-17-3p | 5 | 12 | 1.35221421 |
| bta-miR-17-5p | 578 | 1307 | 156.3159627 |
| bta-miR-1814c | 6 | 11 | 1.622657052 |
| bta-miR-181a | 2955 | 5854 | 799.1585982 |
| bta-miR-181b | 342 | 513 | 92.49145198 |
| bta-miR-181c | 3 | 11 | 0.811328526 |
| bta-miR-181d | 26 | 40 | 7.031513893 |
| bta-miR-182 | 155 | 97 | 41.91864052 |
| bta-miR-183 | 53 | 40 | 14.33347063 |
| bta-miR-1839 | 618 | 906 | 167.1336764 |
| bta-miR-184 | 2 | 6 | 0.540885684 |
| bta-miR-185 | 2596 | 5705 | 702.0696179 |
| bta-miR-186 | 4189 | 5345 | 1132.885065 |
| bta-miR-188 | 3 | 7 | 0.811328526 |
| bta-miR-18a | 7 | 25 | 1.893099894 |
| bta-miR-18b | 1 | 0 | 0.270442842 |
| bta-miR-190a | 2 | 5 | 0.540885684 |
| bta-miR-190b | 1 | 4 | 0.270442842 |
| bta-miR-191 | 10568 | 14981 | 2858.039955 |
| bta-miR-192 | 179799 | 176104 | 48625.35256 |
| bta-miR-193a | 23 | 28 | 6.220185367 |
| bta-miR-193a-3p | 30 | 36 | 8.113285261 |
| bta-miR-193a-5p | 313 | 387 | 84.64860956 |
| bta-miR-193b | 672 | 1861 | 181.7375899 |
| bta-miR-194 | 45458 | 67879 | 12293.79071 |
| bta-miR-195 | 796 | 1458 | 215.2725023 |
| bta-miR-196b | 1 | 0 | 0.270442842 |
| bta-miR-197 | 92 | 141 | 24.88074147 |
| bta-miR-199a-3p | 8777 | 16616 | 2373.676825 |
| bta-miR-199a-5p | 3811 | 7391 | 1030.657671 |
| bta-miR-199b | 1424 | 2090 | 385.1106071 |
| bta-miR-199c | 192 | 501 | 51.92502567 |
| bta-miR-19a | 41 | 54 | 11.08815652 |
| bta-miR-19b | 504 | 758 | 136.3031924 |
| bta-miR-200a | 2323 | 3448 | 628.2387221 |
| bta-miR-200b | 1548 | 1477 | 418.6455195 |

| | | | |
|---|---|---|---|
| bta-miR-200c | 344 | 262 | 93.03233766 |
| bta-miR-204 | 538 | 205 | 145.498249 |
| bta-miR-206 | 1 | 1 | 0.270442842 |
| bta-miR-20a | 2664 | 6312 | 720.4597312 |
| bta-miR-20b | 9 | 19 | 2.433985578 |
| bta-miR-210 | 88 | 208 | 23.7989701 |
| bta-miR-211 | 1 | 0 | 0.270442842 |
| bta-miR-212 | 0 | 3 | 0 |
| bta-miR-21-3p | 0 | 1 | 0 |
| bta-miR-214 | 240 | 515 | 64.90628209 |
| bta-miR-215 | 346 | 687 | 93.57322335 |
| bta-miR-21-5p | 103905 | 135116 | 28100.3635 |
| bta-miR-216b | 2 | 0 | 0.540885684 |
| bta-miR-217 | 1 | 0 | 0.270442842 |
| bta-miR-218 | 794 | 669 | 214.7316166 |
| bta-miR-219 | 0 | 1 | 0 |
| bta-miR-219-3p | 0 | 1 | 0 |
| bta-miR-221 | 230 | 445 | 62.20185367 |
| bta-miR-222 | 80 | 141 | 21.63542736 |
| bta-miR-223 | 126 | 390 | 34.0757981 |
| bta-miR-22-3p | 12755 | 17575 | 3449.49845 |
| bta-miR-224 | 272 | 282 | 73.56045304 |
| bta-miR-22-5p | 285 | 345 | 77.07620998 |
| bta-miR-2284aa | 7 | 6 | 1.893099894 |
| bta-miR-2284ab | 23 | 32 | 6.220185367 |
| bta-miR-2284ac | 7 | 4 | 1.893099894 |
| bta-miR-2284e | 0 | 2 | 0 |
| bta-miR-2284f | 3 | 7 | 0.811328526 |
| bta-miR-2284h-5p | 10 | 15 | 2.70442842 |
| bta-miR-2284j | 2 | 3 | 0.540885684 |
| bta-miR-2284r | 1 | 0 | 0.270442842 |
| bta-miR-2284v | 1 | 0 | 0.270442842 |
| bta-miR-2284w | 38 | 73 | 10.276828 |
| bta-miR-2284x | 1589 | 2878 | 429.733676 |
| bta-miR-2284y | 101 | 138 | 27.31472705 |
| bta-miR-2284z | 12 | 11 | 3.245314104 |
| bta-miR-2285aa | 13 | 16 | 3.515756947 |
| bta-miR-2285ab | 2 | 0 | 0.540885684 |
| bta-miR-2285ad | 2 | 1 | 0.540885684 |
| bta-miR-2285af | 4 | 13 | 1.081771368 |
| bta-miR-2285b | 16 | 22 | 4.327085473 |
| bta-miR-2285c | 1 | 1 | 0.270442842 |
| bta-miR-2285e | 3 | 14 | 0.811328526 |
| bta-miR-2285f | 178 | 200 | 48.13882588 |

| | | | |
|---|---|---|---|
| bta-miR-2285g | 4 | 13 | 1.081771368 |
| bta-miR-2285h | 1 | 1 | 0.270442842 |
| bta-miR-2285i | 3 | 4 | 0.811328526 |
| bta-miR-2285j | 4 | 3 | 1.081771368 |
| bta-miR-2285k | 40 | 63 | 10.81771368 |
| bta-miR-2285l | 1 | 0 | 0.270442842 |
| bta-miR-2285m | 0 | 1 | 0 |
| bta-miR-2285n | 1 | 0 | 0.270442842 |
| bta-miR-2285o | 73 | 96 | 19.74232747 |
| bta-miR-2285p | 7 | 18 | 1.893099894 |
| bta-miR-2285q | 5 | 14 | 1.35221421 |
| bta-miR-2285r | 3 | 0 | 0.811328526 |
| bta-miR-2285s | 3 | 1 | 0.811328526 |
| bta-miR-2285t | 508 | 620 | 137.3849638 |
| bta-miR-2285u | 96 | 133 | 25.96251284 |
| bta-miR-2285v | 0 | 2 | 0 |
| bta-miR-2285w | 2 | 0 | 0.540885684 |
| bta-miR-2285x | 5 | 2 | 1.35221421 |
| bta-miR-2285y | 12 | 11 | 3.245314104 |
| bta-miR-2285z | 5 | 14 | 1.35221421 |
| bta-miR-2299-3p | 4 | 6 | 1.081771368 |
| bta-miR-2299-5p | 4 | 6 | 1.081771368 |
| bta-miR-2310 | 6 | 11 | 1.622657052 |
| bta-miR-2311 | 1 | 0 | 0.270442842 |
| bta-miR-2312 | 2 | 3 | 0.540885684 |
| bta-miR-2316 | 7 | 5 | 1.893099894 |
| bta-miR-2318 | 1 | 0 | 0.270442842 |
| bta-miR-2320-3p | 0 | 3 | 0 |
| bta-miR-2331-3p | 0 | 1 | 0 |
| bta-miR-2332 | 76 | 34 | 20.553656 |
| bta-miR-2335 | 1 | 0 | 0.270442842 |
| bta-miR-2336 | 53 | 40 | 14.33347063 |
| bta-miR-2344 | 1 | 1 | 0.270442842 |
| bta-miR-2349 | 1 | 0 | 0.270442842 |
| bta-miR-2353 | 0 | 2 | 0 |
| bta-miR-2355-3p | 4 | 1 | 1.081771368 |
| bta-miR-2366 | 0 | 1 | 0 |
| bta-miR-2367-3p | 1 | 0 | 0.270442842 |
| bta-miR-2370-3p | 1 | 1 | 0.270442842 |
| bta-miR-2376 | 0 | 1 | 0 |
| bta-miR-2378 | 0 | 1 | 0 |
| bta-miR-2387 | 11 | 23 | 2.974871262 |
| bta-miR-2398 | 0 | 3 | 0 |
| bta-miR-2399-3p | 1 | 4 | 0.270442842 |

| | | | |
|---|---|---|---|
| bta-miR-2399-5p | 8 | 6 | 2.163542736 |
| bta-miR-23a | 977 | 2552 | 264.2226567 |
| bta-miR-23b-3p | 1837 | 3117 | 496.8035008 |
| bta-miR-23b-5p | 0 | 4 | 0 |
| bta-miR-24 | 75 | 75 | 20.28321315 |
| bta-miR-2402 | 0 | 1 | 0 |
| bta-miR-2403 | 1 | 0 | 0.270442842 |
| bta-miR-2404 | 7 | 10 | 1.893099894 |
| bta-miR-2408 | 1 | 1 | 0.270442842 |
| bta-miR-2410 | 1 | 0 | 0.270442842 |
| bta-miR-2411-3p | 16 | 13 | 4.327085473 |
| bta-miR-2411-5p | 4 | 4 | 1.081771368 |
| bta-miR-2415-3p | 1 | 3 | 0.270442842 |
| bta-miR-2416 | 1 | 3 | 0.270442842 |
| bta-miR-2419-3p | 0 | 1 | 0 |
| bta-miR-2419-5p | 24 | 87 | 6.490628209 |
| bta-miR-2422 | 0 | 1 | 0 |
| bta-miR-2424 | 1 | 4 | 0.270442842 |
| bta-miR-2425-5p | 1 | 0 | 0.270442842 |
| bta-miR-2431-3p | 0 | 5 | 0 |
| bta-miR-2431-5p | 16 | 15 | 4.327085473 |
| bta-miR-2435 | 3 | 3 | 0.811328526 |
| bta-miR-24-3p | 4762 | 6408 | 1287.848814 |
| bta-miR-2440 | 2 | 9 | 0.540885684 |
| bta-miR-2443 | 4 | 7 | 1.081771368 |
| bta-miR-2447 | 0 | 1 | 0 |
| bta-miR-2458 | 1 | 0 | 0.270442842 |
| bta-miR-2460 | 0 | 1 | 0 |
| bta-miR-2461-3p | 1 | 1 | 0.270442842 |
| bta-miR-2463 | 2 | 1 | 0.540885684 |
| bta-miR-2467-3p | 1 | 0 | 0.270442842 |
| bta-miR-2468 | 9 | 13 | 2.433985578 |
| bta-miR-2478 | 149 | 160 | 40.29598346 |
| bta-miR-2483-3p | 1 | 0 | 0.270442842 |
| bta-miR-2483-5p | 6 | 4 | 1.622657052 |
| bta-miR-2484 | 161 | 159 | 43.54129757 |
| bta-miR-25 | 3979 | 5486 | 1076.092068 |
| bta-miR-26a | 108417 | 158973 | 29320.60161 |
| bta-miR-26b | 23961 | 28816 | 6480.080938 |
| bta-miR-26c | 108414 | 158956 | 29319.79028 |
| bta-miR-27a-3p | 2123 | 3931 | 574.1501537 |
| bta-miR-27a-5p | 8 | 78 | 2.163542736 |
| bta-miR-27b | 57866 | 89163 | 15649.4455 |
| bta-miR-28 | 393 | 613 | 106.2840369 |

| | | | |
|---|---|---|---|
| bta-miR-2887 | 12 | 15 | 3.245314104 |
| bta-miR-2898 | 265 | 595 | 71.66735314 |
| bta-miR-2904 | 113 | 154 | 30.56004115 |
| bta-miR-2957 | 6151 | 12964 | 1663.493921 |
| bta-miR-296-3p | 27 | 41 | 7.301956735 |
| bta-miR-299 | 4 | 13 | 1.081771368 |
| bta-miR-29a | 6926 | 13045 | 1873.087124 |
| bta-miR-29b | 290 | 420 | 78.42842419 |
| bta-miR-29c | 49 | 101 | 13.25169926 |
| bta-miR-29d-3p | 4 | 2 | 1.081771368 |
| bta-miR-29d-5p | 29 | 46 | 7.842842419 |
| bta-miR-301a | 0 | 3 | 0 |
| bta-miR-30a-5p | 119550 | 142237 | 32331.44177 |
| bta-miR-30b-3p | 27 | 25 | 7.301956735 |
| bta-miR-30b-5p | 3032 | 5323 | 819.9826971 |
| bta-miR-30c | 7486 | 8862 | 2024.535116 |
| bta-miR-30d | 23930 | 30116 | 6471.69721 |
| bta-miR-30e-5p | 10364 | 15670 | 2802.869615 |
| bta-miR-30f | 3218 | 3350 | 870.2850657 |
| bta-miR-31 | 413 | 689 | 111.6928938 |
| bta-miR-3120 | 34 | 74 | 9.195056629 |
| bta-miR-32 | 722 | 1050 | 195.259732 |
| bta-miR-320a | 1000 | 1135 | 270.442842 |
| bta-miR-323 | 1 | 4 | 0.270442842 |
| bta-miR-324 | 0 | 7 | 0 |
| bta-miR-326 | 7 | 33 | 1.893099894 |
| bta-miR-328 | 63 | 116 | 17.03789905 |
| bta-miR-329a | 1 | 2 | 0.270442842 |
| bta-miR-330 | 6 | 4 | 1.622657052 |
| bta-miR-331-3p | 113 | 127 | 30.56004115 |
| bta-miR-331-5p | 48 | 59 | 12.98125642 |
| bta-miR-335 | 48 | 42 | 12.98125642 |
| bta-miR-338 | 5 | 14 | 1.35221421 |
| bta-miR-339a | 2030 | 5156 | 548.9989693 |
| bta-miR-339b | 404 | 1410 | 109.2589082 |
| bta-miR-33a | 4 | 18 | 1.081771368 |
| bta-miR-33b | 0 | 1 | 0 |
| bta-miR-340 | 56 | 72 | 15.14479915 |
| bta-miR-342 | 848 | 1214 | 229.3355301 |
| bta-miR-3431 | 284 | 561 | 76.80576714 |
| bta-miR-3432a | 357 | 549 | 96.54809461 |
| bta-miR-345-3p | 6 | 17 | 1.622657052 |
| bta-miR-345-5p | 7 | 25 | 1.893099894 |
| bta-miR-346 | 1 | 0 | 0.270442842 |

| | | | |
|---|---|---|---|
| bta-miR-34a | 52 | 219 | 14.06302779 |
| bta-miR-34b | 0 | 1 | 0 |
| bta-miR-34c | 9 | 11 | 2.433985578 |
| bta-miR-3533 | 0 | 2 | 0 |
| bta-miR-3578 | 14 | 26 | 3.786199789 |
| bta-miR-3596 | 13275 | 19575 | 3590.128728 |
| bta-miR-3600 | 12755 | 17576 | 3449.49845 |
| bta-miR-3601 | 0 | 2 | 0 |
| bta-miR-3604 | 8774 | 16611 | 2372.865496 |
| bta-miR-361 | 233 | 526 | 63.0131822 |
| bta-miR-362-3p | 2 | 7 | 0.540885684 |
| bta-miR-362-5p | 83 | 145 | 22.44675589 |
| bta-miR-363 | 16 | 31 | 4.327085473 |
| bta-miR-365-3p | 2410 | 4619 | 651.7672493 |
| bta-miR-365-5p | 321 | 502 | 86.8121523 |
| bta-miR-369-3p | 99 | 344 | 26.77384136 |
| bta-miR-369-5p | 14 | 17 | 3.786199789 |
| bta-miR-370 | 58 | 89 | 15.68568484 |
| bta-miR-374a | 460 | 841 | 124.4037073 |
| bta-miR-374b | 1043 | 2527 | 282.0718842 |
| bta-miR-375 | 150 | 134 | 40.56642631 |
| bta-miR-376a | 1 | 1 | 0.270442842 |
| bta-miR-376b | 0 | 2 | 0 |
| bta-miR-376c | 1 | 9 | 0.270442842 |
| bta-miR-376d | 0 | 3 | 0 |
| bta-miR-376e | 12 | 39 | 3.245314104 |
| bta-miR-377 | 2 | 4 | 0.540885684 |
| bta-miR-378 | 621 | 1086 | 167.9450049 |
| bta-miR-378b | 0 | 2 | 0 |
| bta-miR-378c | 45 | 127 | 12.16992789 |
| bta-miR-378d | 5 | 2 | 1.35221421 |
| bta-miR-379 | 617 | 1130 | 166.8632335 |
| bta-miR-380-3p | 37 | 123 | 10.00638516 |
| bta-miR-380-5p | 0 | 2 | 0 |
| bta-miR-381 | 138 | 249 | 37.3211122 |
| bta-miR-382 | 17 | 33 | 4.597528315 |
| bta-miR-3956 | 3 | 4 | 0.811328526 |
| bta-miR-3957 | 2 | 3 | 0.540885684 |
| bta-miR-409a | 54 | 119 | 14.60391347 |
| bta-miR-409b | 170 | 273 | 45.97528315 |
| bta-miR-410 | 7 | 41 | 1.893099894 |
| bta-miR-411a | 1107 | 1726 | 299.3802261 |
| bta-miR-411b | 19 | 52 | 5.138413999 |
| bta-miR-411c-3p | 0 | 1 | 0 |

| | | | |
|---|---|---|---|
| bta-miR-411c-5p | 66 | 120 | 17.84922757 |
| bta-miR-421 | 17 | 34 | 4.597528315 |
| bta-miR-423-3p | 1359 | 2531 | 367.5318223 |
| bta-miR-423-5p | 2647 | 3486 | 715.8622029 |
| bta-miR-424-3p | 1 | 15 | 0.270442842 |
| bta-miR-424-5p | 23 | 151 | 6.220185367 |
| bta-miR-425-3p | 42 | 80 | 11.35859937 |
| bta-miR-425-5p | 745 | 873 | 201.4799173 |
| bta-miR-4286 | 12 | 22 | 3.245314104 |
| bta-miR-429 | 29 | 61 | 7.842842419 |
| bta-miR-431 | 0 | 2 | 0 |
| bta-miR-432 | 23 | 43 | 6.220185367 |
| bta-miR-433 | 9 | 23 | 2.433985578 |
| bta-miR-4449 | 2 | 0 | 0.540885684 |
| bta-miR-449a | 14 | 8 | 3.786199789 |
| bta-miR-450a | 152 | 532 | 41.10731199 |
| bta-miR-450b | 234 | 971 | 63.28362504 |
| bta-miR-451 | 3082 | 8016 | 833.5048392 |
| bta-miR-452 | 84 | 114 | 22.71719873 |
| bta-miR-454 | 24 | 21 | 6.490628209 |
| bta-miR-455-3p | 251 | 273 | 67.88115335 |
| bta-miR-455-5p | 1487 | 1240 | 402.1485061 |
| bta-miR-4680 | 2 | 0 | 0.540885684 |
| bta-miR-483 | 89 | 128 | 24.06941294 |
| bta-miR-484 | 85 | 132 | 22.98764157 |
| bta-miR-485 | 1 | 3 | 0.270442842 |
| bta-miR-486 | 147 | 715 | 39.75509778 |
| bta-miR-487a | 2 | 7 | 0.540885684 |
| bta-miR-487b | 28 | 64 | 7.572399577 |
| bta-miR-488 | 0 | 1 | 0 |
| bta-miR-490 | 6 | 15 | 1.622657052 |
| bta-miR-491 | 6 | 9 | 1.622657052 |
| bta-miR-493 | 42 | 41 | 11.35859937 |
| bta-miR-494 | 156 | 429 | 42.18908336 |
| bta-miR-495 | 87 | 186 | 23.52852726 |
| bta-miR-497 | 111 | 214 | 30.01915547 |
| bta-miR-499 | 157 | 212 | 42.4595262 |
| bta-miR-500 | 61 | 89 | 16.49701336 |
| bta-miR-502a | 9 | 10 | 2.433985578 |
| bta-miR-502b | 0 | 2 | 0 |
| bta-miR-503-5p | 1 | 22 | 0.270442842 |
| bta-miR-504 | 8 | 9 | 2.163542736 |
| bta-miR-505 | 259 | 466 | 70.04469609 |
| bta-miR-532 | 1125 | 1645 | 304.2481973 |

| | | | |
|---|---|---|---|
| bta-miR-539 | 1 | 0 | 0.270442842 |
| bta-miR-542-5p | 0 | 2 | 0 |
| bta-miR-543 | 51 | 73 | 13.79258494 |
| bta-miR-545-3p | 0 | 2 | 0 |
| bta-miR-574 | 321 | 428 | 86.8121523 |
| bta-miR-582 | 3 | 4 | 0.811328526 |
| bta-miR-592 | 46 | 79 | 12.44037073 |
| bta-miR-6119-3p | 6 | 17 | 1.622657052 |
| bta-miR-6119-5p | 151 | 295 | 40.83686915 |
| bta-miR-6120-3p | 34 | 57 | 9.195056629 |
| bta-miR-6122-3p | 1 | 0 | 0.270442842 |
| bta-miR-6123 | 1 | 5 | 0.270442842 |
| bta-miR-628 | 6 | 7 | 1.622657052 |
| bta-miR-6516 | 2 | 5 | 0.540885684 |
| bta-miR-6517 | 20 | 16 | 5.408856841 |
| bta-miR-652 | 62 | 159 | 16.76745621 |
| bta-miR-6520 | 12 | 9 | 3.245314104 |
| bta-miR-6521 | 0 | 1 | 0 |
| bta-miR-6522 | 3 | 12 | 0.811328526 |
| bta-miR-6523a | 3 | 9 | 0.811328526 |
| bta-miR-6524 | 23 | 78 | 6.220185367 |
| bta-miR-6525 | 1 | 1 | 0.270442842 |
| bta-miR-6529a | 78 | 118 | 21.09454168 |
| bta-miR-6529b | 78 | 118 | 21.09454168 |
| bta-miR-6530 | 0 | 1 | 0 |
| bta-miR-6531 | 0 | 2 | 0 |
| bta-miR-6533 | 0 | 4 | 0 |
| bta-miR-6536 | 4 | 3 | 1.081771368 |
| bta-miR-654 | 3 | 10 | 0.811328526 |
| bta-miR-655 | 11 | 46 | 2.974871262 |
| bta-miR-656 | 4 | 8 | 1.081771368 |
| bta-miR-660 | 668 | 1380 | 180.6558185 |
| bta-miR-664a | 2 | 0 | 0.540885684 |
| bta-miR-664b | 194 | 438 | 52.46591136 |
| bta-miR-669 | 1 | 0 | 0.270442842 |
| bta-miR-671 | 12 | 18 | 3.245314104 |
| bta-miR-677 | 23 | 57 | 6.220185367 |
| bta-miR-7 | 3811 | 4223 | 1030.657671 |
| bta-miR-708 | 188 | 709 | 50.8432543 |
| bta-miR-744 | 40 | 55 | 10.81771368 |
| bta-miR-758 | 4 | 5 | 1.081771368 |
| bta-miR-760-3p | 1 | 1 | 0.270442842 |
| bta-miR-767 | 1 | 0 | 0.270442842 |
| bta-miR-769 | 38 | 95 | 10.276828 |

| | | | |
|---|---|---|---|
| bta-miR-7857 | 0 | 3 | 0 |
| bta-miR-7858 | 1 | 2 | 0.270442842 |
| bta-miR-7859 | 17 | 41 | 4.597528315 |
| bta-miR-7860 | 2 | 1 | 0.540885684 |
| bta-miR-7862 | 3 | 3 | 0.811328526 |
| bta-miR-7863 | 0 | 1 | 0 |
| bta-miR-873 | 0 | 1 | 0 |
| bta-miR-874 | 142 | 260 | 38.40288357 |
| bta-miR-877 | 4 | 3 | 1.081771368 |
| bta-miR-885 | 167 | 329 | 45.16395462 |
| bta-miR-92a | 5586 | 15141 | 1510.693716 |
| bta-miR-92b | 11 | 19 | 2.974871262 |
| bta-miR-93 | 1010 | 1848 | 273.1472705 |
| bta-miR-935 | 0 | 1 | 0 |
| bta-miR-9-3p | 1 | 2 | 0.270442842 |
| bta-miR-95 | 92 | 107 | 24.88074147 |
| bta-miR-9-5p | 87 | 98 | 23.52852726 |
| bta-miR-96 | 32 | 16 | 8.654170945 |
| bta-miR-98 | 1535 | 2147 | 415.1297625 |
| bta-miR-99a-3p | 46 | 155 | 12.44037073 |
| bta-miR-99a-5p | 66866 | 74378 | 18083.43108 |
| bta-miR-99b | 1178 | 1705 | 318.5816679 |
| novel_1 | 5 | 23 | 1.35221421 |
| novel_10 | 607 | 508 | 164.1588051 |
| novel_11 | 8 | 14 | 2.163542736 |
| novel_12 | 9 | 10 | 2.433985578 |
| novel_13 | 4 | 8 | 1.081771368 |
| novel_14 | 6 | 5 | 1.622657052 |
| novel_15 | 23 | 38 | 6.220185367 |
| novel_16 | 3 | 8 | 0.811328526 |
| novel_17 | 2 | 7 | 0.540885684 |
| novel_18 | 4 | 4 | 1.081771368 |
| novel_19 | 0 | 5 | 0 |
| novel_2 | 8 | 19 | 2.163542736 |
| novel_20 | 0 | 1 | 0 |
| novel_21 | 1 | 8 | 0.270442842 |
| novel_23 | 29 | 35 | 7.842842419 |
| novel_4 | 1 | 1 | 0.270442842 |
| novel_5 | 8 | 7 | 2.163542736 |
| novel_6 | 5 | 4 | 1.35221421 |
| novel_8 | 73 | 42 | 19.74232747 |
| novel_9 | 22 | 7 | 5.949742525 |

**Table S3 Differentially expressed miRNAs between the bull (BL) and steer liver (SL) tissue**

| sRNA | BL | SL | log2.Fold_change. | p.value | q.value |
|---|---|---|---|---|---|
| bta-miR-339b | 284.765491 | 128.5147083 | 1.1478 | 2.75E-28 | 2.65E-27 |
| bta-miR-450b | 196.1044622 | 74.43673699 | 1.3975 | 4.55E-24 | 3.89E-23 |
| bta-miR-486 | 144.4023589 | 46.7615399 | 1.6267 | 7.25E-21 | 5.19E-20 |
| bta-miR-708 | 143.1905909 | 59.80387416 | 1.2596 | 2.38E-16 | 1.41E-15 |
| bta-miR-204 | 41.40207493 | 171.1408739 | -2.0474 | 8.83E-13 | 4.68E-12 |
| bta-miR-450a | 107.4434335 | 48.35206847 | 1.1519 | 1.15E-11 | 5.69E-11 |
| bta-miR-369-3p | 69.47470135 | 31.49246565 | 1.1415 | 5.57E-08 | 2.42E-07 |
| bta-miR-34a | 44.22953371 | 16.54149711 | 1.4189 | 1.28E-06 | 5.00E-06 |
| bta-miR-424-5p | 30.49616251 | 7.316431414 | 2.0594 | 1.44E-06 | 5.53E-06 |
| bta-miR-27a-5p | 15.75298461 | 2.544845709 | 2.63 | 0.00011991 | 0.00038055 |
| bta-miR-99a-3p | 31.30400788 | 14.63286283 | 1.0971 | 0.00035274 | 0.0010752 |
| bta-miR-380-3p | 24.84124496 | 11.7699114 | 1.0776 | 0.0016106 | 0.0045075 |

**Table S4. GO annotations for the predicted target genes**

| GO_accession | Description | Term_type | Over represented pValue | Corrected pValue |
|---|---|---|---|---|
| GO:0003824 | catalytic activity | molecular_function | 2.09E-08 | 8.58E-05 |
| GO:0016740 | transferase activity | molecular_function | 6.50E-08 | 0.00013312 |
| GO:0005125 | cytokine activity | molecular_function | 1.99E-05 | 0.0037016 |
| GO:0050662 | coenzyme binding | molecular_function | 2.47E-05 | 0.0042091 |
| GO:0005488 | binding | molecular_function | 2.84E-05 | 0.0044761 |
| GO:0016772 | transferase activity, transferring phosphorus-containing groups | molecular_function | 3.54E-05 | 0.0048364 |
| GO:0016773 | phosphotransferase activity, alcohol group as acceptor | molecular_function | 9.13E-05 | 0.0098395 |
| GO:0008009 | chemokine activity | molecular_function | 3.46E-07 | 0.00017717 |
| GO:0042379 | chemokine receptor binding | molecular_function | 3.46E-07 | 0.00017717 |
| GO:0048037 | cofactor binding | molecular_function | 1.00E-06 | 0.000457 |
| GO:0001664 | G-protein coupled receptor binding | molecular_function | 4.67E-06 | 0.0017409 |
| GO:0071702 | organic substance transport | biological_process | 1.79E-07 | 0.00017717 |
| GO:0043412 | macromolecule modification | biological_process | 2.00E-07 | 0.00017717 |
| GO:0033036 | macromolecule localization | biological_process | 2.87E-07 | 0.00017717 |
| GO:0016192 | vesicle-mediated transport | biological_process | 3.19E-06 | 0.0013079 |
| GO:0006464 | cellular protein modification process | biological_process | 8.92E-06 | 0.002609 |
| GO:0036211 | protein modification process | biological_process | 8.92E-06 | 0.002609 |
| GO:0044237 | cellular metabolic process | biological_process | 9.59E-06 | 0.0026203 |
| GO:0008104 | protein localization | biological_process | 1.05E-05 | 0.0026987 |
| GO:0009056 | catabolic process | biological_process | 1.12E-05 | 0.0027081 |
| GO:0015031 | protein transport | biological_process | 1.39E-05 | 0.0030005 |
| GO:0045184 | establishment of protein localization | biological_process | 1.39E-05 | 0.0030005 |

| | | | | |
|---|---|---|---|---|
| GO:0051649 | establishment of localization in cell | biological_process | 1.84E-05 | 0.0037016 |
| GO:0008152 | metabolic process | biological_process | 2.17E-05 | 0.0038605 |
| GO:0051641 | cellular localization | biological_process | 1.92E-05 | 0.0037016 |
| GO:0007264 | small GTPase mediated signal transduction | biological_process | 2.98E-05 | 0.0045154 |
| GO:0006810 | transport | biological_process | 3.46E-05 | 0.0048364 |
| GO:0051234 | establishment of localization | biological_process | 3.46E-05 | 0.0048364 |
| GO:0051179 | localization | biological_process | 4.11E-05 | 0.0054319 |
| GO:0009308 | amine metabolic process | biological_process | 4.75E-05 | 0.0060294 |
| GO:0006576 | cellular biogenic amine metabolic process | biological_process | 4.86E-05 | 0.0060294 |
| GO:0044710 | single-organism metabolic process | biological_process | 5.93E-05 | 0.0069464 |
| GO:0006793 | phosphorus metabolic process | biological_process | 7.98E-05 | 0.0090864 |
| GO:0006796 | phosphate-containing compound metabolic process | biological_process | 9.13E-05 | 0.0098395 |
| GO:0032991 | macromolecular complex | cellular_component | 2.70E-05 | 0.0044277 |
| GO:0005737 | cytoplasm | cellular_component | 5.87E-05 | 0.0069464 |
| GO:0044424 | intracellular part | cellular_component | 6.15E-06 | 0.0020997 |
| GO:0043234 | protein complex | cellular_component | 2.58E-07 | 0.00017717 |

**Table S5 Pathway annotations for the predicted target genes**

| #Term | ID | Input number | Background number | P-Value |
|---|---|---|---|---|
| TNF signaling pathway | bta04668 | 45 | 110 | 0.005420569 |
| Hepatitis B | bta05161 | 54 | 149 | 0.016387937 |
| Lysosome | bta04142 | 43 | 123 | 0.044117533 |
| Vasopressin-regulated water reabsorption | bta04962 | 19 | 45 | 0.04582062 |
| NF-kappa B signaling pathway | bta04064 | 33 | 90 | 0.045973436 |
| Glioma | bta05214 | 25 | 64 | 0.046311877 |
| Endocytosis | bta04144 | 70 | 217 | 0.046698375 |
| Apoptosis | bta04210 | 31 | 84 | 0.04891689 |
| Leishmaniasis | bta05140 | 27 | 72 | 0.055266874 |
| MAPK signaling pathway | bta04010 | 81 | 259 | 0.056330597 |
| Toll-like receptor signaling pathway | bta04620 | 37 | 106 | 0.059100986 |
| Fc gamma R-mediated phagocytosis | bta04666 | 31 | 86 | 0.060004622 |
| Nitrogen metabolism | bta00910 | 9 | 17 | 0.062479383 |
| Proteoglycans in cancer | bta05205 | 64 | 201 | 0.065053749 |
| Chemokine signaling pathway | bta04062 | 60 | 188 | 0.069963679 |
| VEGF signaling pathway | bta04370 | 22 | 58 | 0.071969454 |
| Toxoplasmosis | bta05145 | 40 | 119 | 0.074582525 |
| Glycosaminoglycan biosynthesis - keratan sulfate | bta00533 | 8 | 15 | 0.075034772 |
| Amphetamine addiction | bta05031 | 25 | 69 | 0.081259236 |
| Acute myeloid leukemia | bta05221 | 21 | 56 | 0.083312166 |
| Colorectal cancer | bta05210 | 24 | 66 | 0.08409267 |
| Pancreatic cancer | bta05212 | 24 | 66 | 0.08409267 |

| | | | | |
|---|---|---|---|---|
| Ras signaling pathway | bta04014 | 72 | 234 | 0.084650943 |
| Hepatitis C | bta05160 | 44 | 135 | 0.087467803 |
| Peroxisome | bta04146 | 28 | 80 | 0.088933465 |
| SNARE interactions in vesicular transport | bta04130 | 14 | 34 | 0.089393815 |
| Rap1 signaling pathway | bta04015 | 66 | 214 | 0.092381473 |
| T cell receptor signaling pathway | bta04660 | 36 | 108 | 0.093399909 |
| B cell receptor signaling pathway | bta04662 | 25 | 71 | 0.099217417 |
| mTOR signaling pathway | bta04150 | 22 | 61 | 0.100114383 |
| Alanine, aspartate and glutamate metabolism | bta00250 | 14 | 35 | 0.10289022 |
| Biosynthesis of amino acids | bta01230 | 26 | 75 | 0.105059857 |
| Synaptic vesicle cycle | bta04721 | 23 | 65 | 0.106819158 |
| Ubiquitin mediated proteolysis | bta04120 | 44 | 138 | 0.107790104 |
| AMPK signaling pathway | bta04152 | 40 | 124 | 0.108698969 |
| Neurotrophin signaling pathway | bta04722 | 40 | 124 | 0.108698969 |
| Osteoclast differentiation | bta04380 | 43 | 135 | 0.111784319 |
| Renal cell carcinoma | bta05211 | 23 | 66 | 0.117700784 |
| Prostate cancer | bta05215 | 29 | 87 | 0.121594619 |
| Tuberculosis | bta05152 | 56 | 183 | 0.122445698 |
| Citrate cycle (TCA cycle) | bta00020 | 12 | 30 | 0.124897533 |
| Gastric acid secretion | bta04971 | 25 | 74 | 0.130620344 |
| Focal adhesion | bta04510 | 62 | 206 | 0.131007821 |
| PI3K-Akt signaling pathway | bta04151 | 100 | 346 | 0.132464761 |
| Fatty acid metabolism | bta01212 | 17 | 47 | 0.13391025 |
| Steroid biosynthesis | bta00100 | 9 | 21 | 0.134730358 |
| Oxytocin signaling pathway | bta04921 | 47 | 153 | 0.141088921 |
| Adherens junction | bta04520 | 23 | 68 | 0.141425417 |
| Glycosphingolipid biosynthesis - lacto and neolacto series | bta00601 | 11 | 28 | 0.147921259 |
| Salmonella infection | bta05132 | 27 | 83 | 0.154319991 |
| NOD-like receptor signaling pathway | bta04621 | 18 | 52 | 0.158060618 |
| Long-term potentiation | bta04720 | 22 | 66 | 0.160723427 |
| Fatty acid degradation | bta00071 | 15 | 42 | 0.161518397 |
| Pertussis | bta05133 | 25 | 77 | 0.167337938 |
| Valine, leucine and isoleucine degradation | bta00280 | 16 | 46 | 0.171889148 |
| Adipocytokine signaling pathway | bta04920 | 23 | 71 | 0.18178023 |
| p53 signaling pathway | bta04115 | 23 | 71 | 0.18178023 |
| Fatty acid elongation | bta00062 | 9 | 23 | 0.182239678 |
| Arginine and proline metabolism | bta00330 | 20 | 61 | 0.190369001 |
| Lysine degradation | bta00310 | 18 | 54 | 0.190464671 |
| Phototransduction | bta04744 | 11 | 30 | 0.19107457 |
| Dilated cardiomyopathy | bta05414 | 27 | 86 | 0.19257325 |
| Carbon metabolism | bta01200 | 32 | 104 | 0.192800261 |
| Hypertrophic cardiomyopathy (HCM) | bta05410 | 25 | 79 | 0.194636036 |
| Leukocyte transendothelial migration | bta04670 | 36 | 119 | 0.198752315 |

| | | | | |
|---|---|---|---|---|
| Cocaine addiction | bta05030 | 17 | 51 | 0.199048174 |
| FoxO signaling pathway | bta04068 | 40 | 134 | 0.203294711 |
| Chronic myeloid leukemia | bta05220 | 23 | 73 | 0.211665784 |
| HIF-1 signaling pathway | bta04066 | 31 | 102 | 0.213443633 |
| Pyruvate metabolism | bta00620 | 13 | 38 | 0.217251611 |
| HTLV-I infection | bta05166 | 76 | 270 | 0.223495037 |
| ErbB signaling pathway | bta04012 | 26 | 85 | 0.229740706 |
| beta-Alanine metabolism | bta00410 | 11 | 32 | 0.239153681 |
| Cysteine and methionine metabolism | bta00270 | 13 | 39 | 0.239567675 |
| Dopaminergic synapse | bta04728 | 38 | 130 | 0.243081134 |
| Bacterial invasion of epithelial cells | bta05100 | 23 | 75 | 0.243703302 |
| Vascular smooth muscle contraction | bta04270 | 35 | 119 | 0.244230524 |
| Collecting duct acid secretion | bta04966 | 10 | 29 | 0.251449948 |
| Inflammatory mediator regulation of TRP channels | bta04750 | 31 | 105 | 0.254622254 |
| Adrenergic signaling in cardiomyocytes | bta04261 | 42 | 146 | 0.257119452 |
| Purine metabolism | bta00230 | 50 | 176 | 0.258130157 |
| GnRH signaling pathway | bta04912 | 26 | 87 | 0.260649427 |
| cAMP signaling pathway | bta04024 | 56 | 199 | 0.263085869 |
| Endometrial cancer | bta05213 | 16 | 51 | 0.268362348 |
| Insulin secretion | bta04911 | 25 | 84 | 0.271578142 |
| Cytokine-cytokine receptor interaction | bta04060 | 66 | 238 | 0.275524474 |
| Chagas disease (American trypanosomiasis) | bta05142 | 33 | 114 | 0.277290826 |
| Other types of O-glycan biosynthesis | bta00514 | 10 | 30 | 0.278990702 |
| Histidine metabolism | bta00340 | 8 | 23 | 0.279533194 |
| Epstein-Barr virus infection | bta05169 | 54 | 193 | 0.279676585 |
| Inflammatory bowel disease (IBD) | bta05321 | 21 | 70 | 0.283906237 |
| Estrogen signaling pathway | bta04915 | 29 | 100 | 0.290602625 |
| Folate biosynthesis | bta00790 | 5 | 13 | 0.291205552 |
| Fatty acid biosynthesis | bta00061 | 5 | 13 | 0.291205552 |
| Gap junction | bta04540 | 26 | 89 | 0.293111828 |
| Homologous recombination | bta03440 | 9 | 27 | 0.294638183 |
| Melanoma | bta05218 | 21 | 71 | 0.302539345 |
| Thyroid hormone synthesis | bta04918 | 21 | 71 | 0.302539345 |
| Phagosome | bta04145 | 45 | 161 | 0.303214293 |
| Long-term depression | bta04730 | 18 | 60 | 0.303938843 |
| Melanogenesis | bta04916 | 29 | 101 | 0.306323982 |
| ABC transporters | bta02010 | 13 | 42 | 0.311114505 |
| Ovarian steroidogenesis | bta04913 | 16 | 53 | 0.311527675 |
| Oocyte meiosis | bta04114 | 32 | 113 | 0.317793014 |
| DNA replication | bta03030 | 11 | 35 | 0.318255106 |
| Regulation of actin cytoskeleton | bta04810 | 58 | 212 | 0.322020164 |
| Butanoate metabolism | bta00650 | 9 | 28 | 0.32521204 |
| Circadian entrainment | bta04713 | 28 | 99 | 0.334847829 |

| | | | | |
|---|---|---|---|---|
| Sulfur relay system | bta04122 | 5 | 14 | 0.334922488 |
| Influenza A | bta05164 | 48 | 175 | 0.335788783 |
| Fructose and mannose metabolism | bta00051 | 10 | 32 | 0.336348869 |
| Propanoate metabolism | bta00640 | 8 | 25 | 0.345092617 |
| One carbon pool by folate | bta00670 | 6 | 18 | 0.353450891 |
| Amyotrophic lateral sclerosis (ALS) | bta05014 | 16 | 55 | 0.356415444 |
| Fat digestion and absorption | bta04975 | 13 | 44 | 0.361541999 |
| Ether lipid metabolism | bta00565 | 13 | 44 | 0.361541999 |
| Signaling pathways regulating pluripotency of stem cells | bta04550 | 38 | 139 | 0.365474552 |
| Proximal tubule bicarbonate reclamation | bta04964 | 7 | 22 | 0.367494156 |
| Sphingolipid metabolism | bta00600 | 14 | 48 | 0.368080692 |
| Herpes simplex infection | bta05168 | 52 | 193 | 0.368921538 |
| Axon guidance | bta04360 | 34 | 124 | 0.369992712 |
| Glutathione metabolism | bta00480 | 15 | 52 | 0.373964663 |
| Fanconi anemia pathway | bta03460 | 15 | 52 | 0.373964663 |
| Measles | bta05162 | 38 | 140 | 0.379979513 |
| Tryptophan metabolism | bta00380 | 13 | 45 | 0.387227828 |
| Thyroid cancer | bta05216 | 9 | 30 | 0.38780972 |
| Wnt signaling pathway | bta04310 | 37 | 138 | 0.407315842 |
| Arrhythmogenic right ventricular cardiomyopathy (ARVC) | bta05412 | 19 | 69 | 0.413904982 |
| Pathways in cancer | bta05200 | 86 | 329 | 0.414291235 |
| Arachidonic acid metabolism | bta00590 | 20 | 73 | 0.417193353 |
| Phosphatidylinositol signaling system | bta04070 | 22 | 81 | 0.423132696 |
| ECM-receptor interaction | bta04512 | 23 | 85 | 0.425831058 |
| Cyanoamino acid metabolism | bta00460 | 2 | 5 | 0.428812656 |
| Phenylalanine, tyrosine and tryptophan biosynthesis | bta00400 | 2 | 5 | 0.428812656 |
| Cytosolic DNA-sensing pathway | bta04623 | 17 | 62 | 0.42898514 |
| Aldosterone-regulated sodium reabsorption | bta04960 | 11 | 39 | 0.430481302 |
| Fc epsilon RI signaling pathway | bta04664 | 18 | 66 | 0.432155762 |
| Renin-angiotensin system | bta04614 | 6 | 20 | 0.432750169 |
| Terpenoid backbone biosynthesis | bta00900 | 6 | 20 | 0.432750169 |
| cGMP-PKG signaling pathway | bta04022 | 44 | 167 | 0.433603392 |
| Non-small cell lung cancer | bta05223 | 15 | 55 | 0.445781852 |
| Ubiquinone and other terpenoid-quinone biosynthesis | bta00130 | 3 | 9 | 0.448772831 |
| Galactose metabolism | bta00052 | 9 | 32 | 0.45091685 |
| Pyrimidine metabolism | bta00240 | 27 | 102 | 0.45289249 |
| Non-homologous end-joining | bta03450 | 4 | 13 | 0.459535861 |
| Endocrine and other factor-regulated calcium reabsorption | bta04961 | 12 | 44 | 0.461933495 |
| Nucleotide excision repair | bta03420 | 12 | 44 | 0.461933495 |

| | | | | |
|---|---|---|---|---|
| Platelet activation | bta04611 | 33 | 126 | 0.462247295 |
| GABAergic synapse | bta04727 | 23 | 87 | 0.464213459 |
| Progesterone-mediated oocyte maturation | bta04914 | 23 | 87 | 0.464213459 |
| Glycerophospholipid metabolism | bta00564 | 24 | 91 | 0.465922058 |
| Legionellosis | bta05134 | 15 | 56 | 0.469719282 |
| Viral myocarditis | bta05416 | 18 | 68 | 0.475706005 |
| Base excision repair | bta03410 | 9 | 33 | 0.482162662 |
| Cholinergic synapse | bta04725 | 29 | 112 | 0.490291426 |
| Insulin signaling pathway | bta04910 | 36 | 140 | 0.496603851 |
| RIG-I-like receptor signaling pathway | bta04622 | 20 | 77 | 0.499521691 |
| Taurine and hypotaurine metabolism | bta00430 | 3 | 10 | 0.505753953 |
| Sulfur metabolism | bta00920 | 4 | 14 | 0.507630235 |
| Pentose phosphate pathway | bta00030 | 7 | 26 | 0.511210501 |
| RNA polymerase | bta03020 | 8 | 30 | 0.512124495 |
| Bladder cancer | bta05219 | 10 | 38 | 0.513745675 |
| Regulation of autophagy | bta04140 | 10 | 38 | 0.513745675 |
| Protein processing in endoplasmic reticulum | bta04141 | 43 | 169 | 0.51557491 |
| Bile secretion | bta04976 | 18 | 70 | 0.518791232 |
| Prolactin signaling pathway | bta04917 | 19 | 75 | 0.539972513 |
| Small cell lung cancer | bta05222 | 22 | 87 | 0.540017465 |
| Glycolysis / Gluconeogenesis | bta00010 | 16 | 63 | 0.54019644 |
| Glutamatergic synapse | bta04724 | 29 | 115 | 0.54069429 |
| Linoleic acid metabolism | bta00591 | 9 | 35 | 0.543161769 |
| Circadian rhythm | bta04710 | 8 | 31 | 0.544205728 |
| 2-Oxocarboxylic acid metabolism | bta01210 | 5 | 19 | 0.549976124 |
| Amino sugar and nucleotide sugar metabolism | bta00520 | 12 | 48 | 0.566815924 |
| Primary immunodeficiency | bta05340 | 9 | 36 | 0.572614475 |
| Serotonergic synapse | bta04726 | 29 | 117 | 0.573559876 |
| Glycosaminoglycan biosynthesis - heparan sulfate / heparin | bta00534 | 6 | 24 | 0.583317534 |
| Other glycan degradation | bta00511 | 5 | 20 | 0.58918276 |
| RNA degradation | bta03018 | 18 | 74 | 0.601741493 |
| Prion diseases | bta05020 | 8 | 33 | 0.605585902 |
| Inositol phosphate metabolism | bta00562 | 15 | 62 | 0.607718938 |
| RNA transport | bta03013 | 39 | 160 | 0.612182848 |
| Glyoxylate and dicarboxylate metabolism | bta00630 | 6 | 25 | 0.617690606 |
| Retrograde endocannabinoid signaling | bta04723 | 25 | 104 | 0.626673621 |
| Natural killer cell mediated cytotoxicity | bta04650 | 28 | 117 | 0.638096784 |
| Rheumatoid arthritis | bta05323 | 23 | 97 | 0.646981803 |
| Spliceosome | bta03040 | 31 | 130 | 0.64860845 |
| Morphine addiction | bta05032 | 22 | 93 | 0.649147013 |
| alpha-Linolenic acid metabolism | bta00592 | 6 | 26 | 0.650394582 |
| Ribosome biogenesis in eukaryotes | bta03008 | 20 | 85 | 0.653986513 |
| Intestinal immune network for IgA production | bta04672 | 13 | 56 | 0.657876685 |

| | | | | |
|---|---|---|---|---|
| Mismatch repair | bta03430 | 5 | 22 | 0.661554128 |
| Hematopoietic cell lineage | bta04640 | 22 | 94 | 0.666054846 |
| Type II diabetes mellitus | bta04930 | 11 | 48 | 0.667807083 |
| Transcriptional misregulation in cancer | bta05202 | 41 | 173 | 0.674736752 |
| Selenocompound metabolism | bta00450 | 4 | 18 | 0.676446244 |
| Tight junction | bta04530 | 31 | 132 | 0.677077699 |
| Thyroid hormone signaling pathway | bta04919 | 26 | 112 | 0.687620747 |
| TGF-beta signaling pathway | bta04350 | 18 | 79 | 0.695665764 |
| Cell cycle | bta04110 | 29 | 127 | 0.722258495 |
| Carbohydrate digestion and absorption | bta04973 | 9 | 42 | 0.728075038 |
| Hedgehog signaling pathway | bta04340 | 11 | 51 | 0.732906833 |
| Butirosin and neomycin biosynthesis | bta00524 | 1 | 5 | 0.741811227 |
| Cell adhesion molecules (CAMs) | bta04514 | 35 | 154 | 0.7449281 |
| Glycine, serine and threonine metabolism | bta00260 | 9 | 43 | 0.749875331 |
| Staphylococcus aureus infection | bta05150 | 13 | 61 | 0.756477055 |
| Protein digestion and absorption | bta04974 | 18 | 83 | 0.76067148 |
| Mineral absorption | bta04978 | 10 | 48 | 0.760986585 |
| Glycosphingolipid biosynthesis - ganglio series | bta00604 | 3 | 16 | 0.769702725 |
| Synthesis and degradation of ketone bodies | bta00072 | 2 | 11 | 0.771749461 |
| Phenylalanine metabolism | bta00360 | 4 | 21 | 0.773529531 |
| Salivary secretion | bta04970 | 18 | 84 | 0.775385832 |
| PPAR signaling pathway | bta03320 | 15 | 71 | 0.77686158 |
| Glycosylphosphatidylinositol(GPI)-anchor biosynthesis | bta00563 | 5 | 26 | 0.779551975 |
| Calcium signaling pathway | bta04020 | 42 | 188 | 0.791783651 |
| N-Glycan biosynthesis | bta00510 | 10 | 50 | 0.797907657 |
| Graft-versus-host disease | bta05332 | 8 | 41 | 0.800792258 |
| Pancreatic secretion | bta04972 | 20 | 95 | 0.805831827 |
| Riboflavin metabolism | bta00740 | 2 | 12 | 0.807125602 |
| Amoebiasis | bta05146 | 25 | 117 | 0.808438114 |
| Chemical carcinogenesis | bta05204 | 14 | 69 | 0.813035565 |
| Drug metabolism - other enzymes | bta00983 | 7 | 37 | 0.813410192 |
| Basal cell carcinoma | bta05217 | 11 | 56 | 0.821350534 |
| Protein export | bta03060 | 4 | 23 | 0.824400547 |
| Glycosaminoglycan degradation | bta00531 | 4 | 23 | 0.824400547 |
| Complement and coagulation cascades | bta04610 | 15 | 75 | 0.833181986 |
| mRNA surveillance pathway | bta03015 | 19 | 93 | 0.834451279 |
| Steroid hormone biosynthesis | bta00140 | 11 | 57 | 0.836056523 |
| Viral carcinogenesis | bta05203 | 54 | 246 | 0.847897485 |
| Pantothenate and CoA biosynthesis | bta00770 | 3 | 19 | 0.850877829 |
| Vitamin B6 metabolism | bta00750 | 1 | 8 | 0.868823066 |
| Antigen processing and presentation | bta04612 | 14 | 74 | 0.874307485 |
| Type I diabetes mellitus | bta04940 | 9 | 51 | 0.88106951 |
| Basal transcription factors | bta03022 | 8 | 47 | 0.891460684 |

| | | | | |
|---|---|---|---|---|
| African trypanosomiasis | bta05143 | 6 | 37 | 0.891591342 |
| Glycerolipid metabolism | bta00561 | 10 | 57 | 0.894107862 |
| Drug metabolism - cytochrome P450 | bta00982 | 10 | 57 | 0.894107862 |
| Allograft rejection | bta05330 | 8 | 48 | 0.902579196 |
| Notch signaling pathway | bta04330 | 8 | 48 | 0.902579196 |
| Starch and sucrose metabolism | bta00500 | 8 | 48 | 0.902579196 |
| Malaria | bta05144 | 9 | 53 | 0.903100139 |
| Metabolism of xenobiotics by cytochrome P450 | bta00980 | 10 | 58 | 0.904116714 |
| Ascorbate and aldarate metabolism | bta00053 | 3 | 22 | 0.905884399 |
| Vitamin digestion and absorption | bta04977 | 3 | 22 | 0.905884399 |
| Biosynthesis of unsaturated fatty acids | bta01040 | 3 | 22 | 0.905884399 |
| Hippo signaling pathway | bta04390 | 30 | 151 | 0.907697394 |
| Dorso-ventral axis formation | bta04320 | 3 | 23 | 0.919659518 |
| Glycosphingolipid biosynthesis - globo series | bta00603 | 2 | 17 | 0.92024076 |
| Metabolic pathways | bta01100 | 286 | 1239 | 0.931032276 |
| Pentose and glucuronate interconversions | bta00040 | 4 | 30 | 0.933705812 |
| Nicotinate and nicotinamide metabolism | bta00760 | 4 | 30 | 0.933705812 |
| Maturity onset diabetes of the young | bta04950 | 3 | 25 | 0.941828003 |
| Mucin type O-Glycan biosynthesis | bta00512 | 4 | 31 | 0.942829554 |
| Asthma | bta05310 | 5 | 37 | 0.945561894 |
| Non-alcoholic fatty liver disease (NAFLD) | bta04932 | 30 | 161 | 0.953230563 |
| Nicotine addiction | bta05033 | 5 | 41 | 0.969194468 |
| Taste transduction | bta04742 | 5 | 42 | 0.973389997 |
| Porphyrin and chlorophyll metabolism | bta00860 | 4 | 36 | 0.97345839 |
| MicroRNAs in cancer | bta05206 | 46 | 247 | 0.979833758 |
| Alcoholism | bta05034 | 41 | 224 | 0.980044498 |
| Tyrosine metabolism | bta00350 | 4 | 38 | 0.980690356 |
| Primary bile acid biosynthesis | bta00120 | 1 | 17 | 0.982804154 |
| Retinol metabolism | bta00830 | 7 | 57 | 0.983469305 |
| Autoimmune thyroid disease | bta05320 | 7 | 61 | 0.990532228 |
| Jak-STAT signaling pathway | bta04630 | 26 | 162 | 0.990957434 |
| Cardiac muscle contraction | bta04260 | 10 | 79 | 0.991405167 |
| Aminoacyl-tRNA biosynthesis | bta00970 | 8 | 68 | 0.991899469 |
| Glycosaminoglycan biosynthesis - chondroitin sulfate / dermatan sulfate | bta00532 | 1 | 21 | 0.993031535 |
| Huntington's disease | bta05016 | 31 | 194 | 0.995264476 |
| Alzheimer's disease | bta05010 | 27 | 180 | 0.997350726 |
| Proteasome | bta03050 | 3 | 45 | 0.9983213 |
| Parkinson's disease | bta05012 | 19 | 154 | 0.999580614 |
| Oxidative phosphorylation | bta00190 | 17 | 143 | 0.999598429 |
| Ribosome | bta03010 | 14 | 136 | 0.999903661 |
| Neuroactive ligand-receptor interaction | bta04080 | 42 | 295 | 0.999935141 |
| Systemic lupus erythematosus | bta05322 | 16 | 185 | 0.99999952 |
| Olfactory transduction | bta04740 | 8 | 897 | 1 |
